# Identification and characterization of host factor VCPIP1 as a multi-functional positive regulator of hepatitis B virus

**DOI:** 10.1128/jvi.01581-24

**Published:** 2024-11-04

**Authors:** Ning Kang, Nannan Liu, Mu Liu, Shimei Zhang, Yang Yang, Jia Hou, Dan Tan, Zixiang Gao, Youhua Xie, Zhongliang Shen, Jing Liu

**Affiliations:** 1 Key Laboratory of Medical Molecular Virology (NHC and MOE and CAMS), Department of Medical Microbiology and Parasitology, Shanghai Institute of Infectious Diseases and Biosecurity, School of Basic Medical Sciences, Shanghai Medical College, Fudan University, Shanghai, China; 2 Department of Clinical Laboratory, Children’s Hospital, Fudan University, Shanghai, China; 3 Department of Infectious Diseases, Shanghai Key Laboratory of Infectious Diseases and Biosafety Emergency Response, National Medical Center for Infectious Diseases, Huashan Hospital, Fudan University, Shanghai, China; 4 Shanghai Key Laboratory of Medical Epigenetics, Institutes of Biomedical Sciences, Shanghai Medical College, Fudan University, Shanghai, China; Lerner Research Institute, Cleveland Clinic, Cleveland, Ohio, USA

**Keywords:** HBx, cccDNA, transcriptional regulation, EnI/Xp, YY1

## Abstract

Chronic infection with hepatitis B virus (HBV) remains an important public health challenge. Viral covalently closed circular DNA (cccDNA) persists in infected hepatocytes and serves as the template for transcribing all viral RNA species. HBV regulatory protein X (HBx) interacts with other viral and cellular proteins to play diverse functions in viral life cycle, including modulation of cccDNA transcription activity. Here, we used proximity labeling coupled with proteomic analysis to screen for HBx-interacting host proteins. One of the identified candidates, deubiquitinating enzyme valosin-containing protein-interacting protein 1 (VCPIP1), directly binds HBx and stabilizes HBx by reducing its proteasomal degradation, which corroborated a recent report. VCPIP1-mediated upregulation of HBV transcription, antigen expression, and genome replication was demonstrated using a series of HBV replication and infection models. More importantly, VCPIP1 was found to upregulate HBV in the absence of HBx. Mechanistic studies revealed that VCPIP1 HBx-independently associates with HBV enhancer I/X promoter (EnI/Xp) and positively modulates both its promoter and enhancer activities, partially through promoting the binding of YY1 transcription factor to EnI/Xp. Results presented here expand the recently described role of VCPIP1 in HBV life cycle and establish it as a multi-functional positive regulator of this virus.

**IMPORTANCE:**

Hepatitis B virus (HBV) encodes the regulatory protein HBx that plays crucial roles in viral life cycle and cellular processes through interacting with viral and cellular proteins. Identifying HBx-interacting proteins may reveal novel aspects of host-virus interactions. In this work, proximity labeling coupled with proteomic analysis identified multiple HBx-interacting host factors, and among these, valosin-containing protein-interacting protein 1 (VCPIP1) was confirmed to directly bind HBx and reduce its proteasomal degradation, corroborating a recent report. In addition to upregulating HBx-expressing HBV, we showed that VCPIP1 also positively regulates mutant HBV lacking HBx expression. This novel HBx-independent function of VCPIP1 was shown to involve its association with one viral promoter/enhancer element, which upregulated the latter’s promoter and enhancer activities. These results establish VCPIP1 as a positive regulator of HBV that acts through multiple, diverse mechanisms and might also contribute toward revealing novel cellular functions of VCPIP1.

## INTRODUCTION

Chronic infection with hepatitis B virus (HBV) is an established risk factor for liver cirrhosis and hepatocellular carcinoma (1). The World Health Organization (WHO) estimated that in 2022, 254 million people were chronically infected with HBV, with 1.1 million related deaths and 1.2 million new infections each year (2).

HBV has a partially double-stranded ~3.2 kb relaxed circular DNA (rcDNA) genome (3). Upon entering the hepatocyte via the interaction between viral envelope protein and host Na^+^ taurocholate co-transporting polypeptide (NTCP) receptor (4, 5), the rcDNA genome is ultimately converted into covalently closed circular DNA (cccDNA) in the nucleus (3). HBV cccDNA exists as an episomal, histone-bound minichromosome (6) and serves as the template for all viral transcription, which is driven by host RNA Pol II (7) interacting with four viral promoters: core promoter (Cp), X promoter (Xp), and surface protein promoter 1 and 2 (Sp1 and Sp2) (3). In addition, two enhancer elements, enhancer I and II (EnI and EnII), are located immediately upstream of and overlapping with Xp and Cp, respectively, and regulate the activities of both proximal and distal promoters (8). Cp-initiated transcription generates 3.5 kb pregenomic RNA encoding both core antigen (HBcAg) and polymerase (Pol) and 3.5 kb preCore RNA-encoding e antigen (HBeAg). Transcription driven by Sp1 and Sp2 produces 2.4 kb and 2.1 kb mRNAs encoding large, middle, and small surface antigens (L/M/S-HBsAg), while Xp is responsible for transcribing the 0.7 kb mRNA encoding X protein [HBV regulatory protein X (HBx)]. HBV cccDNA is highly stable, and the nuclear cccDNA pool can be replenished by re-routing cytoplasmic progeny rcDNA-containing capsids to the nucleus (3). Current therapeutic options for chronic hepatitis B (CHB) have minimal effects on cccDNA load in infected hepatocytes (9, 10). “Functional cure,” wherein cccDNA transcription is under sustained suppression, has been adopted as a more realistic, surrogate goal of CHB treatment (11).

Transcription of cccDNA is regulated at epigenetic and transcriptional levels by both host and viral factors. A myriad of cellular proteins has been shown to affect cccDNA histone modification, chromatin structure, and/or enhancer/promoter activities (12). On the other hand, HBx plays a vital role in the regulation of multiple aspects of the viral life cycle and host-virus interactions, including modulation of cccDNA transcription (13), to the extent that HBx is indispensable for the initiation and maintenance of HBV infection (14). Due to the lack of a direct DNA-binding motif, HBx-related regulation invariably involves interaction with other host or viral protein(s). For example, “structural maintenance of chromosomes” (Smc) complex Smc5/6 downregulates the transcription activity of cccDNA, while HBx binds the adaptor component (DDB1, damage-specific DNA-binding protein 1) of the cullin4-RING E3 ligase (CRL4) complex and hijacks CRL4^DDB1^ E3 ligase to ubiquitinylate Smc5/6, leading to the latter’s degradation and consequently relief of its restriction on cccDNA (15, 16). Screening and characterizing HBx-interacting host factors, therefore, would potentially provide new insights into mechanisms of HBx-mediated regulation of viral and host targets.

In this work, we used proximity labeling coupled with proteomic analysis to identify potential HBx-related host proteins. Multiple candidates were obtained and, one of these, deubiquitinating enzyme VCPIP1 (valosin-containing protein-interacting protein 1), was confirmed to bind HBx directly. We characterized the binding between VCPIP1 and HBx and noted the stabilizing effect of such binding on HBx protein, which was recently reported by another group (17). By using a series of HBV replication and infection models, we extensively characterized VCPIP1-mediated upregulation of HBV transcription, antigen expression, and genome replication. More importantly, we observed that VCPIP1 was also able to HBx-independently upregulate HBV, and mechanistic studies of this effect revealed that VCPIP1 indirectly binds a defined element in EnI/Xp and positively modulates its promoter and enhancer activities, partially by promoting the binding of YY1 transcription factor to this element in EnI/Xp.

## MATERIALS AND METHODS

### Cell culture

Human liver cancer cell lines Huh7 and HepG2, HepG2-derived HepAD38 stably transfected with Tet-off inducible 1.1-fold HBV genome (GenBank accession MF967563.1, genotype D) (18), and HEK293T cell lines were cultured in Dulbecco’s modified Eagle medium containing 2 mM L-glutamine, 1:100 penicillin/streptomycin, and 10% fetal bovine serum (all from Thermo Fisher Scientific, China). HepG2 cells stably expressing human NTCP (HepG2-NTCP)(4) were additionally supplemented with 2.5% dimethyl sulfoxide (Sigma, China) and 2 µg/mL puromycin (Thermo). Huh7 and HepG2 cells transducted with recombinant lentivirus carrying VCPIP1-targeting short hairpin RNA (shRNA; Huh7/HepG2-shVCPIP1) were also supplemented with 2 µg/mL puromycin. Primary human hepatocytes (PHHs) were purchased from Liver Biotech, China, and cultured in vendor-supplied media. All cells were cultured at 37°C with 5% CO_2_.

### Plasmids, short interfering RNAs (siRNAs), and transfection

Chemically synthesized coding sequences of Turbo biotin ligase (19) without or with N-terminally fused HBx coding sequences (derived from KR232337) were cloned downstream of FLAG tag in pCMV-N-FLAG (Beyotime, China) to obtain pFLAG-Turbo and pFLAG-HBx-Turbo expression plasmids. Human VCPIP1 (NP_079330.2) and YY1 (NP_003394.1) open reading frames (ORFs) were cloned downstream of the HA tag in pCMV3 (Sino Biological, China), downstream of the FLAG tag in pCMV-N-FLAG, and upstream of the 3×FLAG tag in pcDNA3.1 (YouBio, China) to create pHA-VCPIP1, pFLAG-VCPIP1, and pYY1−3×FLAG, respectively.

Precursor recombinant cccDNA plasmid (prcccDNA, V01460.1, genotype D) and Cre recombinase expression plasmid (pCre) (20), 1.3-fold HBV genome plasmid (p1.3HBV, strain B200, KR232337, genotype B) (21), and cytomegalovirus (CMV) promoter-driven 1.1-fold HBV genome plasmid (pCMV-1.1HBV, genotype C) (22) have been previously described. HBV (strain B200) promoter/enhancer-driven luciferase reporter plasmids pEnII/Cp-Luc, pEnI/Xp-Luc, pSp1-Luc, pSp2-Luc, and positive control plasmid pGL3-CMV were constructed using pGL3-basic vector (Promega, China) as previously described (23, 24). EnI/Xp sequences were amplified and cloned using the NovoRec plus One step PCR Cloning Kit (Novoprotein, China) downstream of luciferase polyA signal in pEnII/Cp-Luc, pSp1-Luc, and pSp2-Luc to create pEnII/Cp-Luc-EnI/Xp, pSp1-Luc-EnI/Xp, and pSp2-Luc-EnI/Xp, respectively.

HBx expression by p1.3HBV and pCMV-1.1HBV was obliterated by mutating CAA to TAA at the eighth amino acid using KOD-plus mutagenesis kit (TOYOBO, Japan) to create p1.3HBV(X*^null^*) and pCMV-1.1HBV(X*^null^*), respectively. Ovarian tumor (OTU) and Ubl domains were similarly deleted from pHA-VCPIP1 to create pHA-VCPIP1ΔOTU and pHA-VCPIP1ΔUbl, respectively. Reporter plasmids for EnI/Xp promoter with serially overlapping 80 nt deletions were also constructed using the KOD-plus mutagenesis kit. VCPIP1 ORF was cloned into pCDH-puro (Addgene, USA), and shRNA sequences targeting VCPIP1 were cloned into pLKO.1-puro (Addgene) to construct the VCPIP1 and shVCPIP1 expression plasmids, respectively. Primers used for mutagenesis and homologous recombination, as well as shRNA sequences, are listed in Table S1.

HEK293T cells were transfected using polyethyleneimine (Sigma), and Huh7, HepG2, and their derived cells were transfected using Turbofect transfection reagent (Thermo) by following manufacturers’ instructions. Chemically synthesized siRNAs targeting YY1 (GenePharma, China) were transfected using Lipofectamine 3000 (Thermo), according to the manufacturer’s instructions. SiRNA sequences are listed in Table S1.

### Proximity labeling and proteomic analysis

Labeling was performed by culturing Huh7 cells transfected with pFLAG-Turbo or pFLAG-HBx-Turbo in media supplemented with 50 µM biotin (Sangon, China) for 30 min. Labeled and unlabeled cells were lysed in RIPA buffer (Sangon), and cleared cell lysates were incubated with Dynabeads M-280 Streptavidin (Thermo) to capture biotinylated proteins. Confirmation of labeling was performed by analyzing captured proteins in Western blot using horseradish peroxidase (HRP)-conjugated streptavidin (Thermo). Lysates from labeled cells in duplicates were sent for commercial trypsin digestion and liquid chromatography - tandem mass spectrometry (LC-MS/MS) analysis (Oebiotech, China) on nanoflow ultra high performance liquid chromatography (UHPLC) (Easy-nLC 1200, Thermo) coupled to hybrid quadrupole Orbitrap (Q Exactive, Thermo). Raw MS data were processed for protein identification and iBAQ quantification in MaxQuant (23) (version 2.1.3.0; parameters used are listed in Table S2). For the analysis of enriched biotinylated protein(s), a published pipeline specially designed for analyzing proximity labeling proteomic data was used with minimal modification (24).

### Co-immunoprecipitation, DNA pull-down, chromatin immunoprecipitation, and *in vitro* translation

Biotinylated double-stranded DNA probes were chemically synthesized (TsingKe, China). FLAG-tagged proteins, HA-tagged proteins, and biotinylated proteins or DNA were captured using anti-FLAG M2 magnetic beads (Sigma), anti-HA magnetic beads (Thermo), and Dynabeads M-280 Streptavidin beads, respectively, as previously described (25). Chromatin immunoprecipitation (ChIP) assay was performed using SimpleChIP Plus Enzymatic Chromatin IP Kit (#9005, Cell Signaling Technology) according to manufacturer’s instructions. *In vitro* translation was performed using the TnT T7 Quick Coupled Transcription/Translation System kit (Promega) according to the manufacturer’s instructions.

### Immunofluorescence and Western blot

Immunofluorescence detection of endogenous VCPIP1 was performed using anti-VCPIP1 antibody as previously described (26), and cell nuclei were stained with 4,6-diamidino-2-phenylindole (Sangon, China). Western blot was performed according to standard procedure as previously described(27). Antibodies used in this work are listed in Table S3.

### Viruses and infection

Recombinant lentiviruses were prepared by transfecting HEK293T cells with pLKO.1 or pCDH plasmids and helper plasmids pSPAX2 and pMD2.G (Addgene) at the ratio of 4:3:1. Media were changed at 8 hours post-transfection, and supernatants were collected 3 days later, filtered through 0.45 µm filter, and used for infection in the presence of 8 µg/ml polybrene (Sigma). Media were changed at 12 hours post-infection, and stable transductants were selected by supplementing culture media with 2 µg/mL puromycin.

HBV virions were prepared by concentrating HepAD38 culture supernatants and quantified as previously described (22). HepG2-NTCP and PHH cells were infected at 1,000:1 multiplicity of infection (MOI) for 12 and 16 hours, respectively, before changing into fresh media.

### Measurement of HBV antigen and nucleic acid markers

HBsAg and HBeAg in culture media were detected using enzyme linked immunosorbent assay (ELISA) (Kehua, China). Intracellular capsid-associated HBV DNA was extracted and detected in Southern blot using digoxigenin (DIG)-labeled full-length HBV probes as previously described (21). HBV (r)cccDNA was extracted using the Hirt method as previously described (25). Cellular RNA was extracted using NucleoZOL (TaKaRa, China) and analyzed using NorthernMax Kit (Thermo) and DIG-labeled DNA probes following the manufacturer’s instructions. Intracellular HBV capsids were extracted by lysing cells in 1% NP40 and electrophoresed in 1% agarose gel followed by transfer to nitrocellulose membrane and detection using anti-HBcAg antibody (DAKO).

### Quantitative real-time PCR

Reverse transcription was performed using PrimeScript RT reagent with gDNA eraser (TaKaRa), and cDNA was quantified using LightCycler 480 SYBR Green I Master (Roche) on CFX Connect (Bio-rad) according to manufacturers’ instructions. Primers for quantitative real-time PCR (qPCR) are listed in Table S1.

### Reporter assay

Enhancer/promoter activity was measured using the Dual-Luciferase Assay System (Promega), following manufacturers’ instructions.

### Transcription factor binding sites prediction

Potential binding sites of human transcription factors were predicted using PROMO (28) (https://alggen.lsi.upc.es/cgi-bin/promo_v3/promo/promoinit.cgi?dirDB=TF_8.3) with a threshold of 3% maximum matrix dissimilarity.

### Data analysis

GraphPad Prism 9 was used for statistical comparison and significance calculation using *t*-test or two-way ANOVA test when applicable.

## RESULTS

### Proximity labeling identified VCPIP1 as an HBx-binding host factor

**Fig 1:**
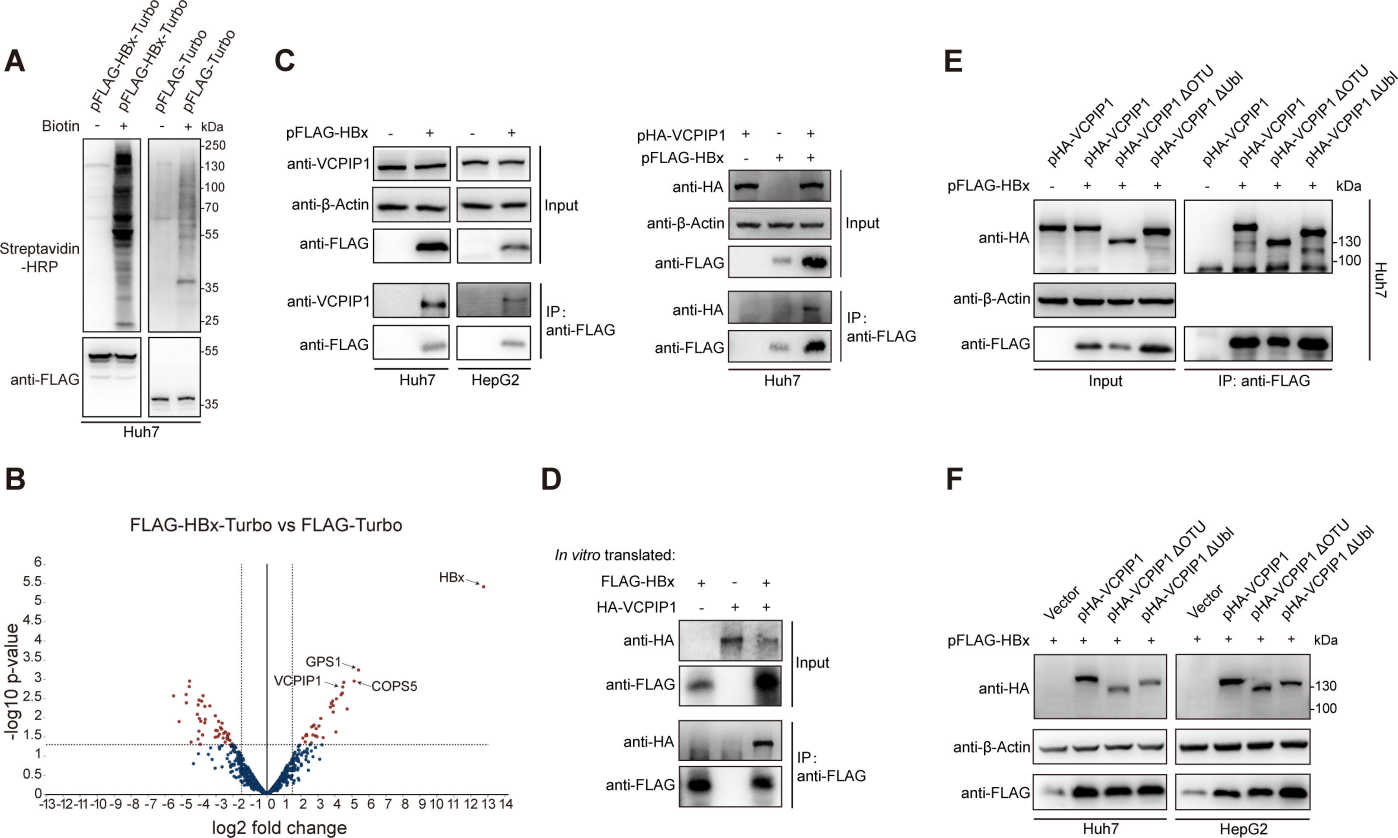
Identification of VCPIP1 as an HBx-binding host factor through proximity labeling. (**A**) Confirmation of TurboID-mediated protein labeling. Huh7 cells in 24-well plate were transfected with 1 µg plasmid expressing TurboID biotin ligase (pFLAG-Turbo) or HBx-TurboID fusion protein (pFLAG-HBx-Turbo). Culture media were supplemented with 50 µM biotin 24 hours later, and cells were further cultured for 30 min. Biotinylated proteins in cell lysates were captured by streptavidin-conjugated beads and analyzed in Western blot using HRP-conjugated streptavidin. (**B**) Captured biotinylated proteins as analyzed in (**A**) (right lanes) were subjected to LC-MS/MS analysis as described in Materials and Methods, and statistically enriched biotinylated proteins in pFLAG-HBx-Turbo-transfected cells compared against pFLAG-Turbo-transfected cells were obtained (shown here as a volcano plot with selected proteins indicated and as full list in Table S4). FC, fold change in protein iBAQ quantities calculated by MaxQuant software. (**C**) Co-IP of endogenous (left) and exogenous (right) VCPIP1 with HBx. Cells in 6 cm dishes were transfected with 6 µg pFLAG-HBx or vector control (left), or 3 µg pFLAG-HBx plus 3 µg pHA-VCPIP1 or vector control (right). Forty-eight hours later, FLAG-tagged HBx in cell lysates was captured using anti-FLAG magnetic beads, and co-immunoprecipitated proteins were analyzed in Western blot using indicated antibodies. (**D**) HBx and VCPIP1 were translated *in vitro* and binding between them was analyzed similarly as in (**C**). (**E**) Effects of deletion of OTU or Ubl domain on VCPIP1 association with HBx in co-IP as performed in (**C**). (**F**) Effects of VCPIP1 and its deletion mutants on HBx protein level. Cells in 12-well plate were co-transfected with 1 µg of indicated VCPIP1 plasmid and 1 µg pFLAG-HBx, and 48 hours later, cell lysates were analyzed in Western blot.

In order to screen HBx-interacting host factors, we adopted the TurboID-based proximity labeling technique (19) and expressed the TurboID biotin ligase alone (Turbo) or as a fusion protein with HBx (HBx-Turbo) in Huh7 cells. Culture of transfected cells in biotin-supplemented media for 30 min resulted in marked increase in biotinylated proteins, indicating successful labeling (Fig. 1A). Biotinylated proteins in cell lysates were then precipitated using streptavidin-coupled magnetic beads and subjected to LC-MS/MS analysis. After protein identification and quantification using the MaxQuant software (23), a published proximity labeling data-processing pipeline (24) was used to compare and identify significantly enriched biotinylated proteins in HBx-Turbo-expressing cells vs Turbo-expressing cells (Fig. 1B; Table S4). HBx expectedly came out as the most enriched biotinylated protein due to self-biotinylation by HBx-Turbo, while a total of 30 host factors were identified, including, most notably, damage-specific DNA-binding protein 1 (DDB1), cullin 4B (CUL4B), and COP9 signalosome complex (CSN) subunits (COPS) 1–6 and 7A. Cullin 4A/4B binds the RING domain-containing protein RBX1 to form the cullin4-RING E3 ligase (CRL4), while DDB1 is the adaptor between cullin 4 in CRL4 and various substrate receptors that recruit protein substrates for CRL4^DDB1^-mediated ubiquitination (29). It has long been established that HBx binds DDB1 to hijack CRL4^DDB1^ E3 ligase for targeted ubiquitination of host restriction factors leading to their degradation (15, 16). On the other hand, CSN-mediated deNEDDylation of CRL is a key mechanism for inhibiting CRL activity in the absence of receptor-mediated substrate binding (29). In other words, the identification of significant biotinylation of DDB1 and CSN components in HBx-Turbo-expressing cells confirmed both the successful labeling of HBx-proximal host proteins by HBx-Turbo and the validity of the data-processing pipeline.

Due to the small number of remaining identified host factors after the removal of DDB1 and CSN components, gene ontology analysis failed to generate meaningful results (data not shown). Among these factors, VCPIP1 is a member of the OTU domain-containing family of deubiquitinases (DUB) and was subjected to further analysis due to HBx’s link to cellular ubiquitination pathways (15, 16). Co-immunoprecipitation (Co-IP) assay using cell lysates demonstrated the association of HBx with both endogenously and exogenously expressed VCPIP1 (Fig. 1C Fig. S1), while co-IP using *in vitro*-translated proteins confirmed a direct binding between HBx and VCPIP1 (Fig. 1D).

The 1,222 amino acid VCPIP1 contains an OTU domain (a.a. 164–360) conferring DUB activity, and a ubiquitin regulatory X (UBX)-like (Ubl) domain (a.a. 775–849) with potential protein-binding functions (30). Previously, VCPIP1’s OTU domain was shown to be essential for its interaction with DNA-dependent metalloprotease SPRN1 (31). We, therefore, tested whether these domains were involved in VCPIP1-HBx binding. Deletion of either OTU or Ubl domain from VCPIP1, however, did not affect its association with HBx in co-IP assay (Fig. 1E). These data indicated that HBx-binding most likely does not involve its OTU or Ubl domains.

### VCPIP1 binding improves HBx protein stability

In addition to the binding between VCPIP1 and HBx, an unexpected and interesting observation was also made from the co-IP results: overexpression of VCPIP1 was associated with apparently higher expression of HBx protein in the input samples (Fig. 1C, right and Fig. S1B). This effect was also observed when VCPIP1 mutants lacking OTU or Ubl domains were exogenously expressed (Fig. 1E) and was more clearly observable when overexpressed VCPIP1 and mutants were compared against endogenous VCPIP1 (Fig. 1F). Conversely, when endogenous VCPIP1 expression was knocked down through RNA interference, lower HBx expression was observed (Fig. S2A). Further analysis revealed that VCPIP1 overexpression/knockdown did not affect HBx mRNA levels in transfected cells (Fig. S2B). When protein translation was inhibited by cycloheximide, VCPIP1 overexpression was associated with a slower decrease of pre-existing HBx protein level, while knockdown of endogenous VCPIP1 resulted in quicker HBx degradation (Fig. S2C). Moreover, treatment of Huh7 cells with proteasome inhibitor MG132, but not lysosome inhibitor chloroquine, rescued HBx from degradation associated with the knockdown of endogenous VCPIP1 expression (Fig. S2D). Collectively, these results showed that VCPIP1 positively regulates HBx protein stability by slowing down its degradation through the proteasomal pathway. Recently, Wu et al. reported that VCPIP1 is capable of stabilizing HBx by forming a tertiary complex with HBx and 26S proteasome regulatory subunit 6A (PSMC3) to prevent HBx degradation by 20S proteasome in a ubiquitination-independent manner (17). Our data were consistent with this report and corroborated VCPIP1-mediated stabilizing effect on HBx protein.

### VCPIP1 enhances HBV transcription, antigen expression, and genome replication

**Fig 2:**
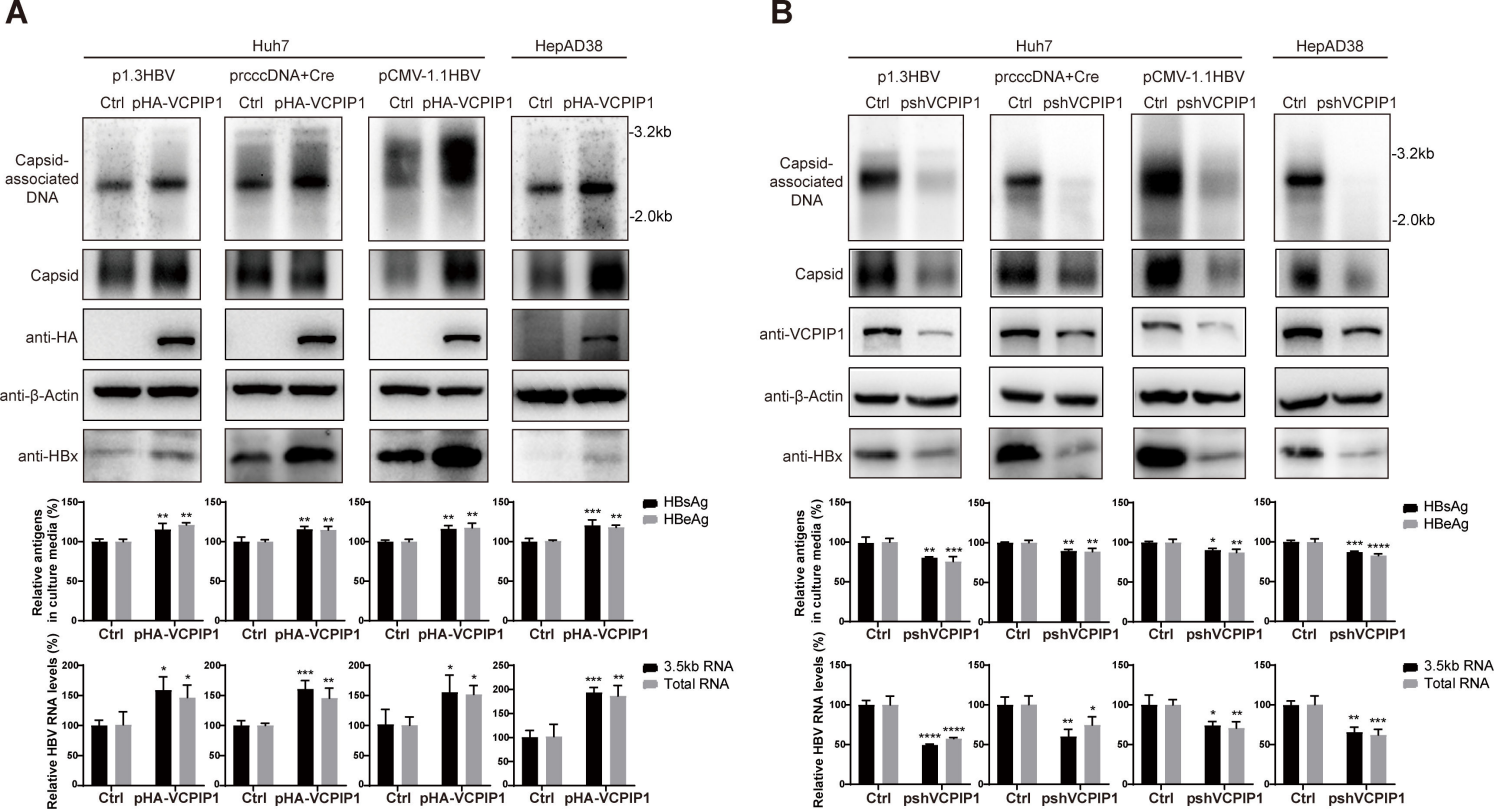
VCPIP1 upregulates HBV transcription, antigen expression, and genome replication in HBV replication models. Huh7 cells in six-well plate were transfected with 2 µg p1.3HBV, 2 µg pCMV-1.1HBV, or 1 µg prcccDNA plus 1 µg pCre, along with 1 µg pHA-VCPIP1 (**A**) or pshVCPIP1 (**B**). HepAD38 cells in 6 cm dishes were transfected with 6 µg pHA-VCPIP1 (**A**) or pshVCPIP1 (**B**). After 4 days, capsid-associated HBV DNA was analyzed in Southern blot (top). Intracellular HBV capsids were analyzed using agarose gel electrophoresis followed by protein blotting using HBcAg antibody. Intracellular endogenous/exogenous VCPIP1 and HBx were analyzed in Western blot. Secreted HBsAg and HBeAg were measured using ELISA, and intracellular HBV RNA (total and 3.5 kb RNAs) was measured using RT-qPCR (bottom). Relative antigen levels are calculated by normalizing against vector-transfected control group. Relative RNA levels are calculated by first normalizing against 18S rRNA measured in parallel followed by a second normalization against control group. Experiments were repeated three times. Group means and SDs were presented, and significances were calculated using two-way ANOVA. *, *P* < 0.05; **, *P* < 0.01; ***, *P* < 0.001; ****, *P* < 0.0001.

Given that HBx played a critical positive regulatory role in HBV life cycle, we tested the effects of VCPIP1 using four different HBV replication cell models. Huh7 cells were transfected with either 1.3-fold over-length HBV genome plasmid (p1.3HBV), CMV promoter-driven 1.1-fold over-length HBV genome (pCMV-1.1HBV), or recombinant cccDNA precursor plasmid (prcccDNA) (20) plus Cre recombinase expression plasmid to initiate HBV gene expression and genome replication from episomal plasmids and rcccDNA, respectively. Additionally, HepAD38, a HepG2-derived cell line stably transfected with HBV genome driven by Tet-off inducible CMV promoter, was cultured without Tet inducer and used as a model of chromosomally initiated HBV transcription. In all these models, exogenous overexpression of VCPIP1 resulted in the elevation of viral transcription, increased production of secreted HBsAg and HBeAg, and more importantly, higher levels of intracellular capsids and capsid-associated progeny HBV DNA (Fig. 2A). Such upregulation of viral activities was accompanied by higher HBx protein levels (Fig. 2A). Conversely, knockdown of endogenous VCPIP1 expression in these models resulted in inhibition of HBV transcription and replication, along with moderately decreased production of secreted antigens, and reduced HBx protein levels (Fig. 2B). Clearly, upregulation of HBx protein level by VCPIP1-mediated stabilizing is also effective in these models of HBV replication and correlated with upregulated HBV gene expression and genome replication. Moreover, in cells with stable knockdown of endogenous VCPIP1 expression (Huh7-shVCPIP1 and HepG2-shVCPIP1), we found that exogenous VCPIP1 expression rescued, to varying degrees, HBV replication after pCMV-1.1HBV and p1.3HBV transfection (Fig. S3A). Additionally, Southern blot showed that the deletion of the OTU domain of VCPIP1 weakened its upregulation of replication from HBV promoter-driven replicons (p1.3HBV and prcccDNA/Cre), indicating that the OTU domain and/or its DUB activity contributes toward this effect (Fig. S3B).

**Fig 3:**
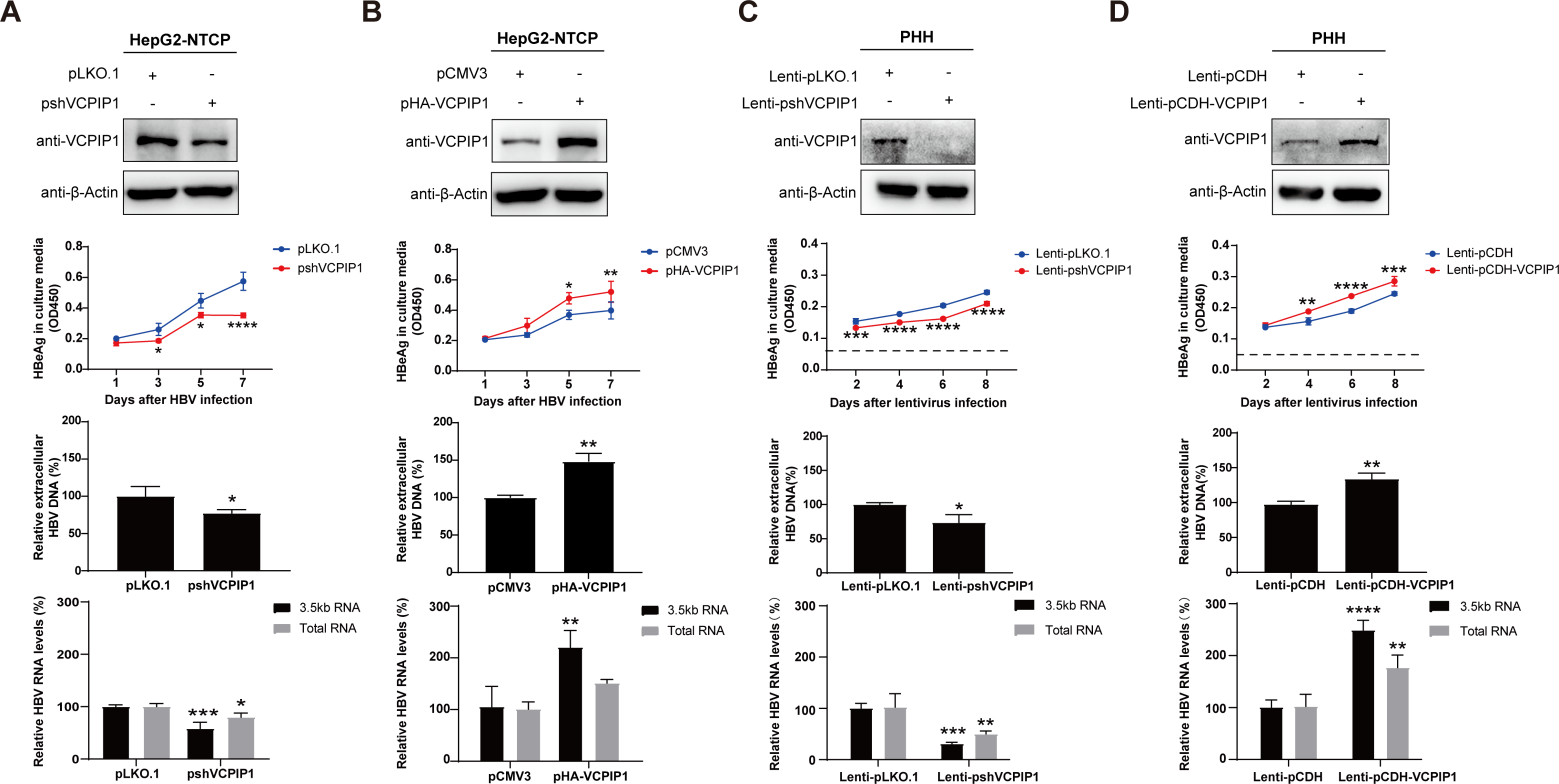
VCPIP1 upregulates HBV transcription, antigen expression, and genome replication in HBV-infected HepG2-NTCP and PHH cells. HepG2-NTCP cells were transfected with 1 µg pshVCPIP1 (**A**), pHA-VCPIP1 (**B**), or corresponding empty vector controls as indicated, and 8 hours later, infected with HBV at MOI of 1,000:1. Cells were changed into fresh media 12 hours later (day 0), and culture media samples were collected at days 1, 3, and 5. On day 7, cells were harvested for VCPIP1 protein analysis by Western blot (top), and intracellular HBV RNA (total and 3.5 kb RNAs) was measured using RT-qPCR (bottom). HBV antigens (day 1/3/5/7) and progeny viral DNA (day 7) in culture media were measured using ELISA and qPCR, respectively (middle). PHHs were first infected with HBV at MOI of 1,000:1 and changed into fresh media 16 hours later. Seventy-two hours later, cells were transduced with shVCPIP1-expressing (Lenti-pLKO.1-shVCPIP1) (**C**) or VCPIP1-expressing (**D**) recombinant lentiviruses, or corresponding control lentiviruses as indicated. Culture media were sampled at days 2, 4, 6, and 8 post lentivirus infection, and cells were harvested at day 8. Media and cell samples were similarly analyzed as described above. Longer exposure times were used for VCPIP1 Western blot analyses in (**A**) and (**C**) to illustrate reduced, but not entirely absent, endogenous VCPIP1 expression. Experiments were repeated three times. Group means and SDs were presented, and significances were calculated using *t*-test or two-way ANOVA, *, *P* < 0.05; **, *P* < 0.01; ***, *P* < 0.001; ****, *P* < 0.0001.

HBx has been shown to play an indispensable role in HBV infection of primary human hepatocytes (14). We first tested the effects of VCPIP1 on HBV infection using HepG2 cells stably expressing HBV receptor NTCP (HepG2-NTCP). As shown in Fig. 3A, knockdown of endogenous VCPIP1 expression in HepG2-NTCP cells resulted in slower production of secreted HBeAg post-infection and reduced viral transcription and progeny virus production at 7 days post-infection. Expectedly, overexpression of exogenous VCPIP1 in HepG2-NTCP displayed the opposite effect (Fig. 3B). We then modulated endogenous VCPIP1 protein levels in HBV-infected PHHs using transduction with recombinant lentiviruses expressing VCPIP1 or VCPIP1-targeting shRNA. Effects of changed VCPIP1 levels on HBV in PHH are similar compared to those observed in HepG2-NTCP cells (Fig. 3C and D). Although HBx expression in these models was too low to be detected, these results agreed with data obtained using HBV replication models (Fig. 2) and confirmed a positive regulatory role played by VCPIP1 in HBV life cycle. In the recent report by Wu et al., overexpression of VCPIP1 and/or PSMC3 was also shown to cause increased HBV transcription, antigen secretion, and progeny virus production in infected HepG2-NTCP cells.

### VCPIP1 positively regulates HBV in the absence of HBx

**Fig 4:**
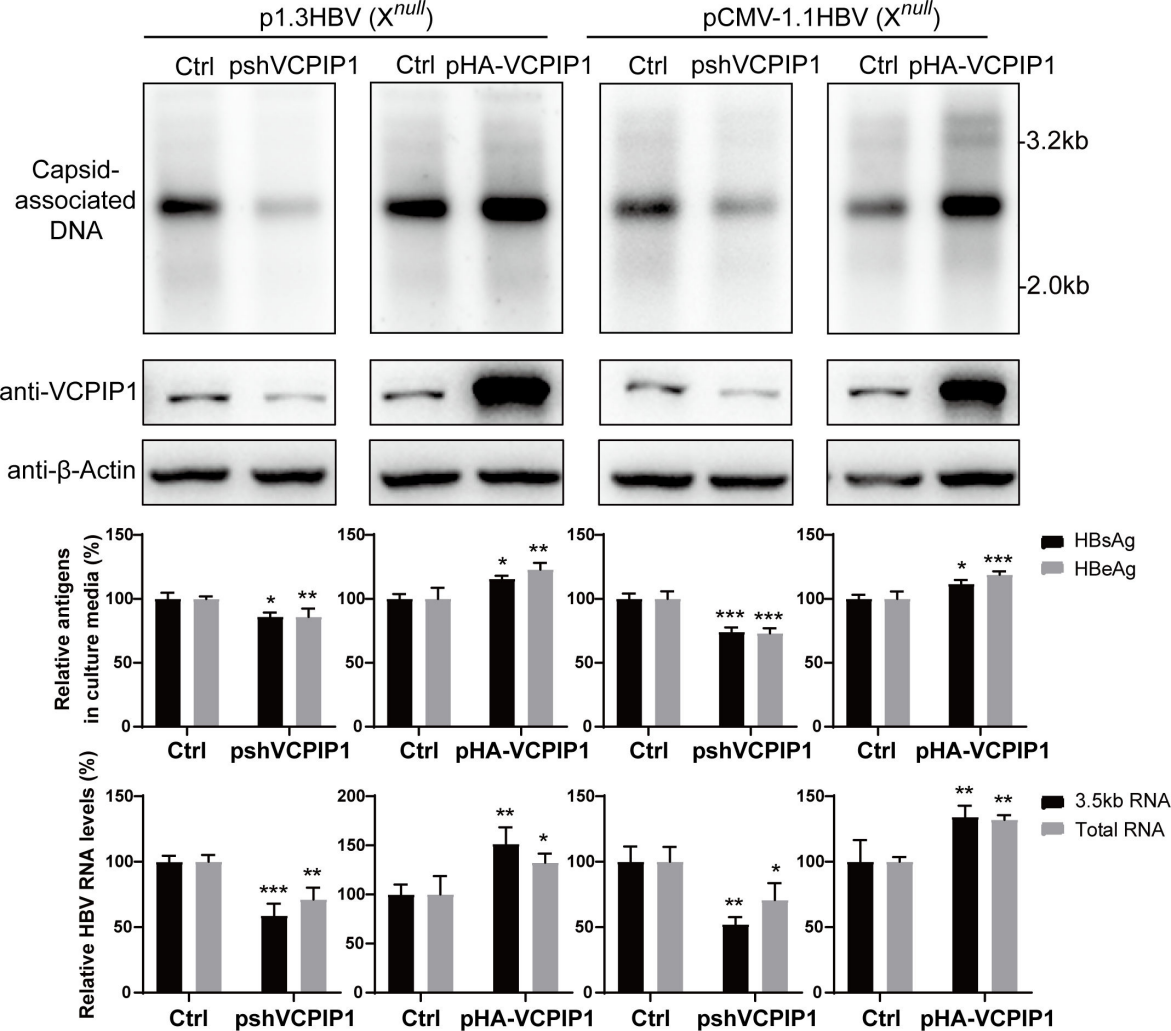
VCPIP1 positively regulates HBV in the absence of HBx. Huh7 cells in six-well plate were transfected with 2 µg p1.3HBV(X*^null^*) or pCMV-1.1HBV(X*^null^*) along with 1 µg pshVCPIP1 or pHA-VCPIP1 as indicated. After 4 days, capsid-associated HBV DNA was analyzed in Southern blot (top). Intracellular VCPIP1 was analyzed in Western blot. Secreted HBsAg and HBeAg were measured using ELISA, and intracellular HBV RNA (total and 3.5 kb RNAs) was measured using RT-qPCR (bottom). Relative antigen and RNA levels are calculated by normalizing against vector-transfected control group. Experiments were repeated at least three times. Group means and SDs were presented, and significances were calculated using two-way ANOVA with GraphPad Prism 9, *, *P* < 0.05; **, *P* < 0.01; ***, *P* < 0.001.

In addition to its stabilizing effect on HBx protein, we wondered whether VCPIP1 might also affect other aspects of HBV life cycle. Since HBx is essential for virion infection, we tested this possibility using mutant HBV genomes with prematurely terminated HBx ORF in replication models. In Huh7 cells transfected with p1.3HBV(X*^null^*) or pCMV-1.1HBV(X*^null^*), knockdown of endogenous VCPIP1 expression reduced viral transcription, production of secreted viral antigens, as well as progeny viral genome replication, whereas exogenous expression of VCPIP1 displayed the opposite effects (Fig. 4). These observations were similar to those made using HBx-expressing HBV genomes (Fig. 2) and demonstrated that at least for these two models, VCPIP1 also positively upregulates HBV life cycle through HBx-unrelated mechanism(s).

### VCPIP1 upregulates EnI/Xp promoter and enhancer activities

**Fig 5:**
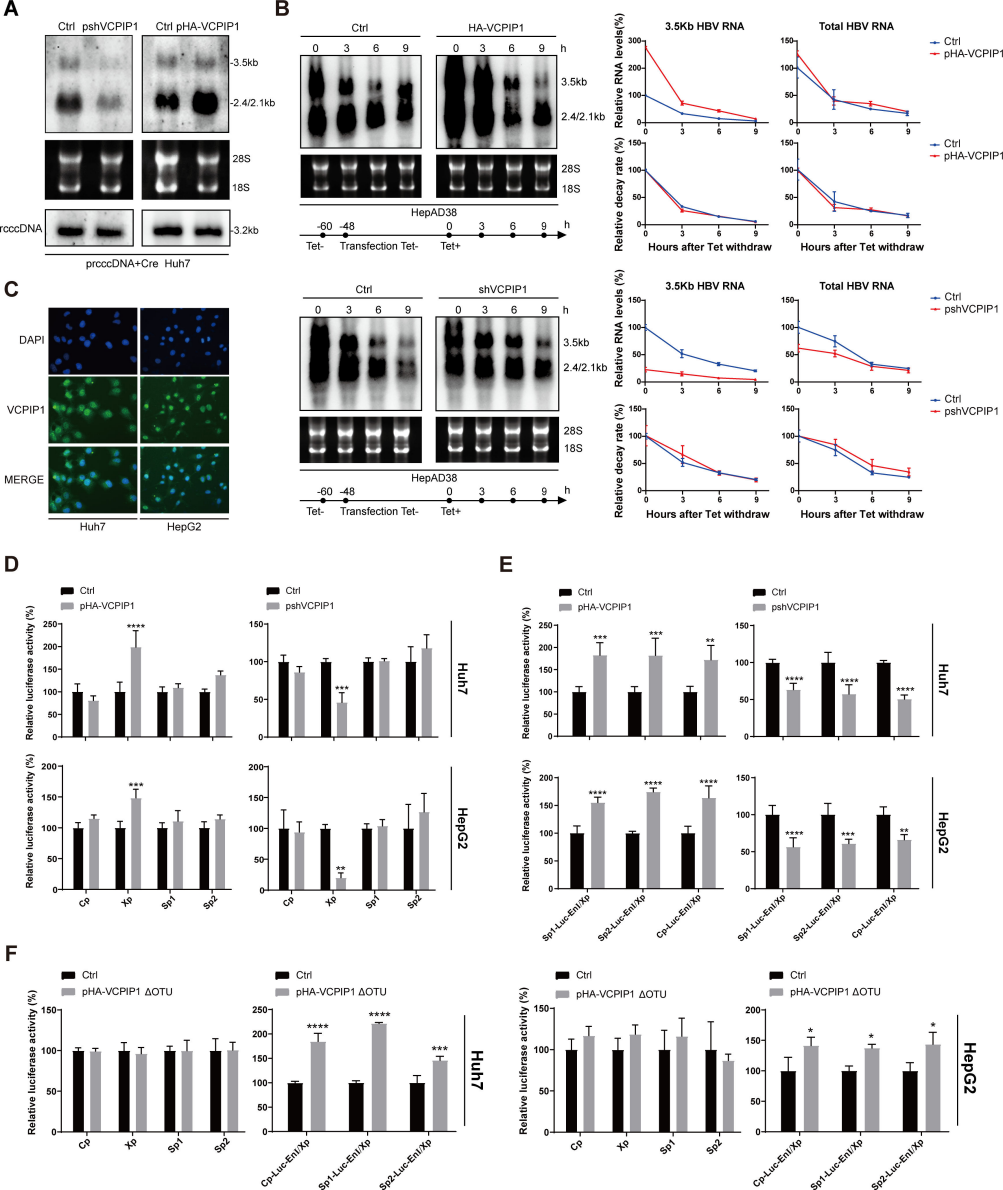
VCPIP1 upregulates HBV transcription and activates EnI/Xp promoter and enhancer activities. (**A**) VCPIP1’s effect on transcription from HBV rcccDNA. Huh7 cells cultured in six-well plate were transfected with 1 µg prcccDNA, 1 µg pCre, along with 1 µg pshVCPIP1 or pHA-VCPIP1 as indicated. After 4 days, intracellular HBV RNA was analyzed using Northern blot. Nuclear rcccDNA was extracted and analyzed using Southern blot after denaturation at 85℃ and linearization by EcoRI digestion. (**B**) VCPIP1’s effect on HBV RNA stability. HepAD38 cells in six-well plate were transfected with 3 µg pshVCPIP1 or pHA-VCPIP1 as indicated. Two days later, cells were changed into fresh media containing 1 µg/mL dox to shut off HBV transcription, and intracellular RNA extracted at 0, 3, 6, and 9 hours post dox treatment was analyzed in northern blot (left) and RT-qPCR (right). Relative RNA levels are calculated by normalizing against vector-transfected control group (top), and relative RNA decay rates were calculated by further normalizing against values at 0 hour (bottom). (**C**) Subcellular localization of endogenous VCPIP1 in Huh7 and HepG2 cells was analyzed using immunofluorescence with anti-VCPIP1 antibody. VCPIP1’s effect on HBV enhancer/promoter-driven transcription (**D**) and EnI/Xp-mediated enhancement of upstream HBV enhancer/promoter (**E**) was analyzed using reporter assay. Cells in 12-well plate were co-transfected with 0.6 µg indicated luciferase reporter plasmids and 0.1 µg Renilla luciferase expression plasmid, along with 1 µg pHA-VCPIP1 or pshVCPIP1 as indicated. Relative luciferase activities were measured 2 days later using dual-luciferase reporter assay and calculated by normalizing against vector-transfected control group. (**F**) pHA-VCPIP1ΔOTU was used instead of pHA-VCPIP1, and the same reporter assays as (**D**) and (**E**) were performed. Experiments were repeated at least three times. Group means and SDs were presented, and significances were calculated using two-way ANOVA with GraphPad Prism 9, *, *P* < 0.05; **, *P* < 0.01; ***, *P* < 0.001; ****, *P* < 0.0001.

To probe mechanism(s) underlying HBx-independent upregulation of HBV by VCPIP1, we first evaluated its effects on cccDNA using the rcccDNA model. While Southern blot showed that nuclear rcccDNA levels were unaffected by changes in VCPIP1 protein level, Northern blot revealed that VCPIP1 was associated with higher levels of both 3.5 Kb and 2.4/2.1 Kb HBV (Fig. 5A), consistent with results described earlier (Fig. 2). We then used the HepAD38 model to test VCPIP1’s effects on HBV RNA stability. Transfected HepAD38 cells were changed from Tet-free to Tet-supplemented media to suppress transcription from chromosomally integrated HBV genome, and samples were collected at 3-hour intervals for RNA analysis. Both Northern blot and RT-qPCR showed that similar to earlier results (Fig. 2), VCPIP1 overexpression was associated with higher HBV RNA levels before the Tet-off induction, and the shutdown of viral transcription resulted in a rapid decrease in HBV RNA levels in all cases (Fig. 5B). Nevertheless, the speed of RNA degradation was comparable and not affected by VCPIP1 overexpression or knockdown (Fig. 5B). These data reconfirmed VCPIP1-mediated upregulation of HBV RNA levels and demonstrated that such upregulation works at transcriptional, but not post-transcriptional, stage.

In both Huh7 and HepG2 cells, endogenous VCPIP1 was found predominantly in the nucleus but also in the cytoplasm (Fig. 5C). We, therefore, went on to test possible effects of VCPIP1 on HBV promoter/enhancer elements in the absence of HBx co-expression. Reporter assay revealed that among the four HBV promoter/enhancer elements, only EnI/Xp was affected markedly by VCPIP1. VCPIP1 overexpression was associated with higher EnI/Xp promoter activity, whereas knockdown of endogenous VCPIP1 suppressed its promoter activity (Fig. 5D; Fig. S4A). Furthermore, when EnI/Xp enhancer activity was analyzed by placing it downstream of reporter ORF (Fig. S4A), reporter expression driven by the other three promoter/enhancer elements was similarly upregulated by VCPIP1 (Fig. 5E). Such upregulation of EnI/Xp promoter and enhancer activities was consistent with VCPIP1’s effects on HBV RNA levels (Fig. 2, 3 and 5A and B) and, in particular, could explain the HBx-independent effects (Fig. 4). Interestingly, deletion of the OTU domain obliterated VCPIP1-mediated enhancement of EnI/Xp promoter activity but did not affect its upregulation of EnI/Xp enhancer activity (Fig. 5F). Clearly, these two activities were upregulated by VCPIP1 through different mechanism(s).

### Mapping of the VCPIP1-responsive element within EnI/Xp

**Fig 6:**
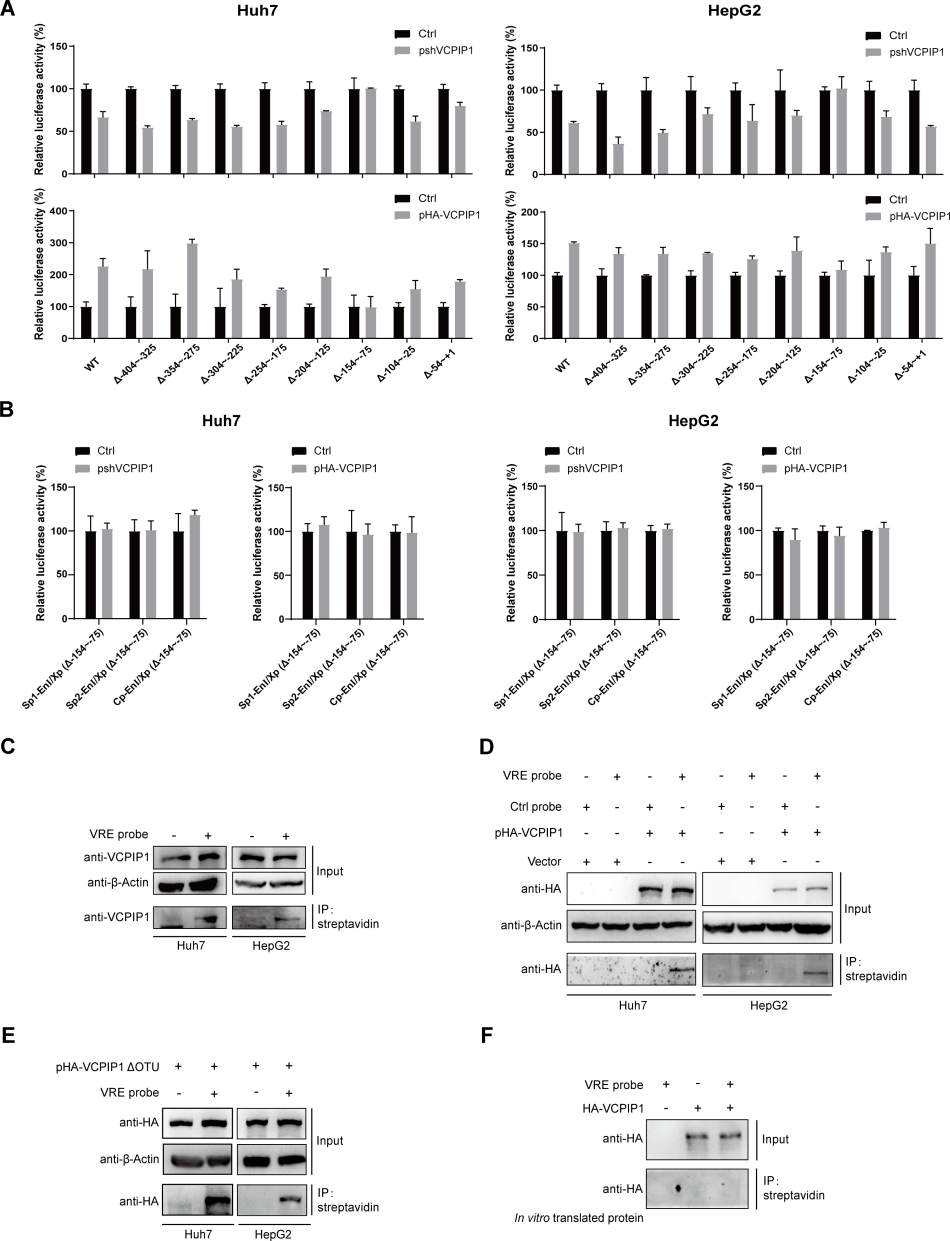
Mapping of the VRE within EnI/Xp. VCPIP1’s effect on EnI/Xp promoter (**A**) and enhancer (**B**) activities was analyzed using EnI/Xp reporter plasmids containing indicated deletions. Cells in 12-well plate were co-transfected with 0.6 µg indicated luciferase reporter plasmids and 0.1 µg Renilla luciferase expression plasmid, along with 1 µg pHA-VCPIP1 or pshVCPIP1 as indicated. Relative luciferase activities were measured 2 days later using dual-luciferase reporter assay and calculated by normalizing against vector-transfected control group. Experiments were repeated at least three times. Group means and SDs were presented, and significances were calculated using two-way ANOVA test. Binding between the mapped VRE at −154 to −75 nt of EnI/Xp was examined by using chemically synthesized biotin-labeled VRE double-stranded DNA probe to capture endogenous (**C**) and exogenous (**D**) VCPIP1 in cell lysates in DNA pull-down assay using streptavidin beads. Captured VCPIP1 was detected using Western blot. A similar assay was performed to examine VRE binding with mutant VCPIP1 lacking OTU (**E**) and *in vitro* translated VCPIP1 (**F**).

To delineate VCPIP1-mediated upregulation of EnI/Xp, we introduced serially overlapping 80 nt deletions (Fig. S4B) and used EnI/Xp promoter activity assay to map potential VCPIP1-responsive element(s) (VRE). As shown in Fig. 6A, most deletion mutants displayed responsiveness to VCPIP1 overexpression/knockdown, while the deletion of −154 to −75 nt resulted in nearly total loss of responsiveness. Moreover, this deletion also caused irresponsiveness in EnI/Xp enhancer activity assay (Fig. 6B). To test whether VCPIP1 binds to this VRE, we synthesized biotinylated double-stranded DNA (dsDNA) probe using original or shuffled VRE sequences and used the probes to capture VCPIP1 in cell lysates. Both endogenous VCPIP1 (Fig. 6C) and exogenously expressed VCPIP1 (Fig. 6D) could be captured by VRE probe but not by the control probe (Fig. S5A). However, the deletion of the OTU domain reduced but did not obliterate VCPIP1’s association with VRE probe (Fig. 6E; Fig. S5B). VCPIP1 does not contain any known DNA-binding domain or motif, and accordingly, no direct binding between *in vitro* translated VCPIP1 and VRE probe could be observed (Fig. 6F). It would appear that although VCPIP1 largely acts through VRE to affect EnI/Xp promoter and enhancer activities, this process requires other cellular factor(s).

### VCPIP1 promotes binding of YY1 transcription factor to VRE in EnI/Xp

**Fig 7:**
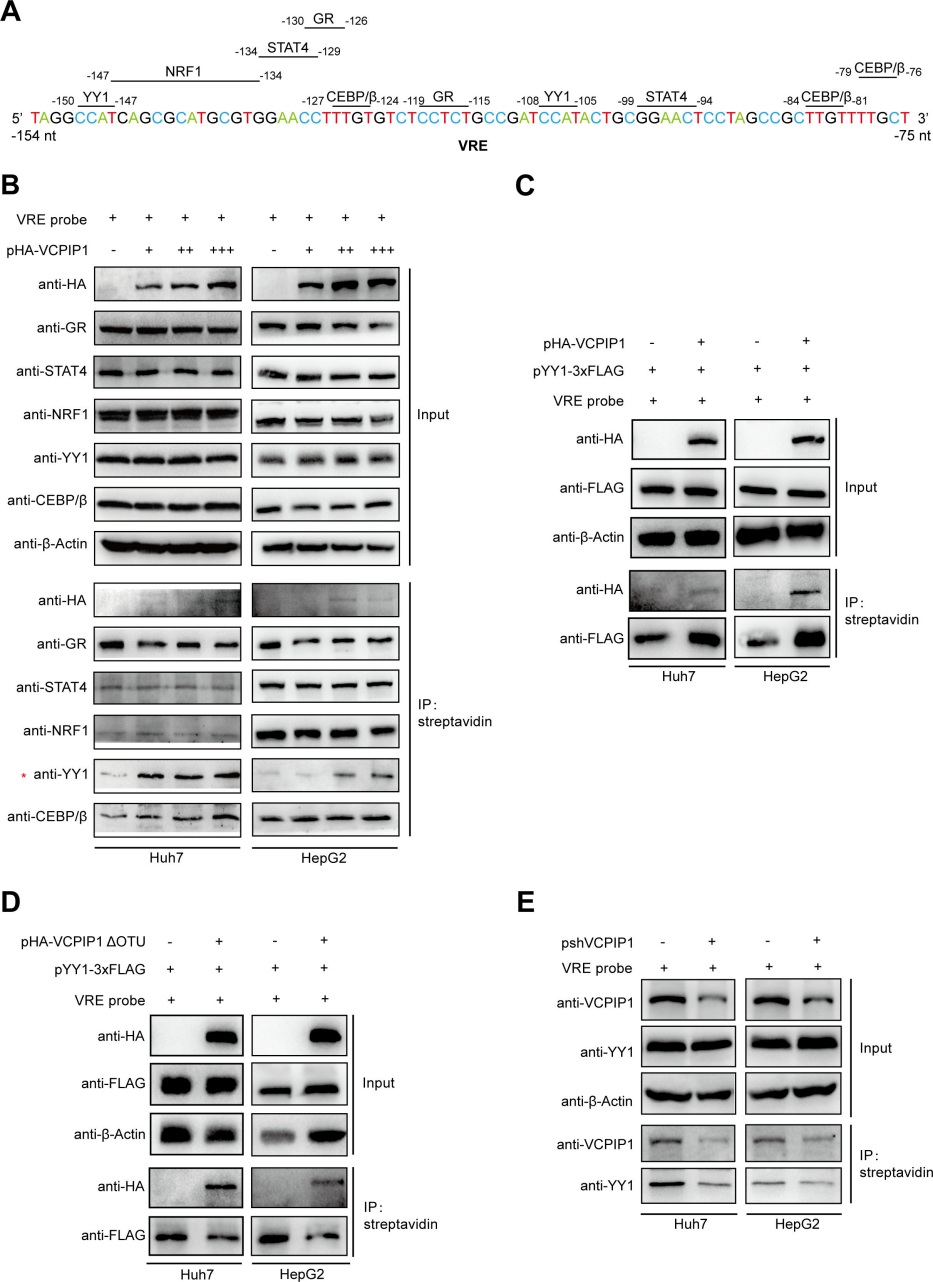
VCPIP1 promotes binding of transcription factor YY1 to VRE. (**A**) VRE sequences with predicted transcription factor binding sites indicated. (**B**) VCPIP1’s effect on transcription factors’ binding to VRE. Cells were transfected with 0, 2, 4, and 6 µg of pHA-VCPIP1, respectively, and 2 days later, cell lysates were incubated with biotin-labeled VRE dsDNA probe. Captured transcription factors that were predicted to bind VRE by PROMO website were analyzed in Western blot. The effect of overexpression of VCPIP1 (**C**) or mutant VCPIP1 lacking OTU (**D**) on exogenously expressed YY1’s binding to VRE probe and the effect of knockdown of endogenous VCPIP1 on binding of endogenous YY1 to VRE were similarly analyzed (**E**).

To identify factor(s) involved in VCPIP1-mediated regulation via VRE, we searched for potential binding sites of known DNA-binding transcription factors within the VRE element using the PROMO website (28) and obtained five candidates (GR, STAT4, NRF1, YY1, and CEBP/β; Fig. 7A). Binding sites for these factors within VRE are highly conserved across HBV genotypes (Fig. S6A), and all of the five candidates could be captured from cell lysates by VRE probe, but not by a control probe, indicating capability of VRE binding (Fig. 7B; Fig. S5A). VCPIP1 overexpression did not affect mRNA or protein levels of these factors (Fig. S6B&C) or affect their binding to VRE with the exception of YY1: increased VCPIP1 expression resulted in more YY1 bound and captured by VRE probe from cell lysates (Fig. 7B). VCPIP1-enhanced binding to VRE was also observed for exogenously expressed YY1 (Fig. 7C) and apparently required the OTU domain of VCPIP1 (Fig. 7D). Conversely, VCPIP1 knockdown reduced YY1 binding to VRE (Fig. 7E). Interestingly, although both VCPIP1 and YY1 could be captured by VRE probe, no association between the two was observable in co-IP assay (Fig. S7). In the context of HBV genome, ChIP assay demonstrated YY1 occupancy on rcccDNA VRE, and such occupancy was increased by VCPIP1 overexpression and reduced by knockdown of endogenous VCPIP1 (Fig. S8).

**Fig 8:**
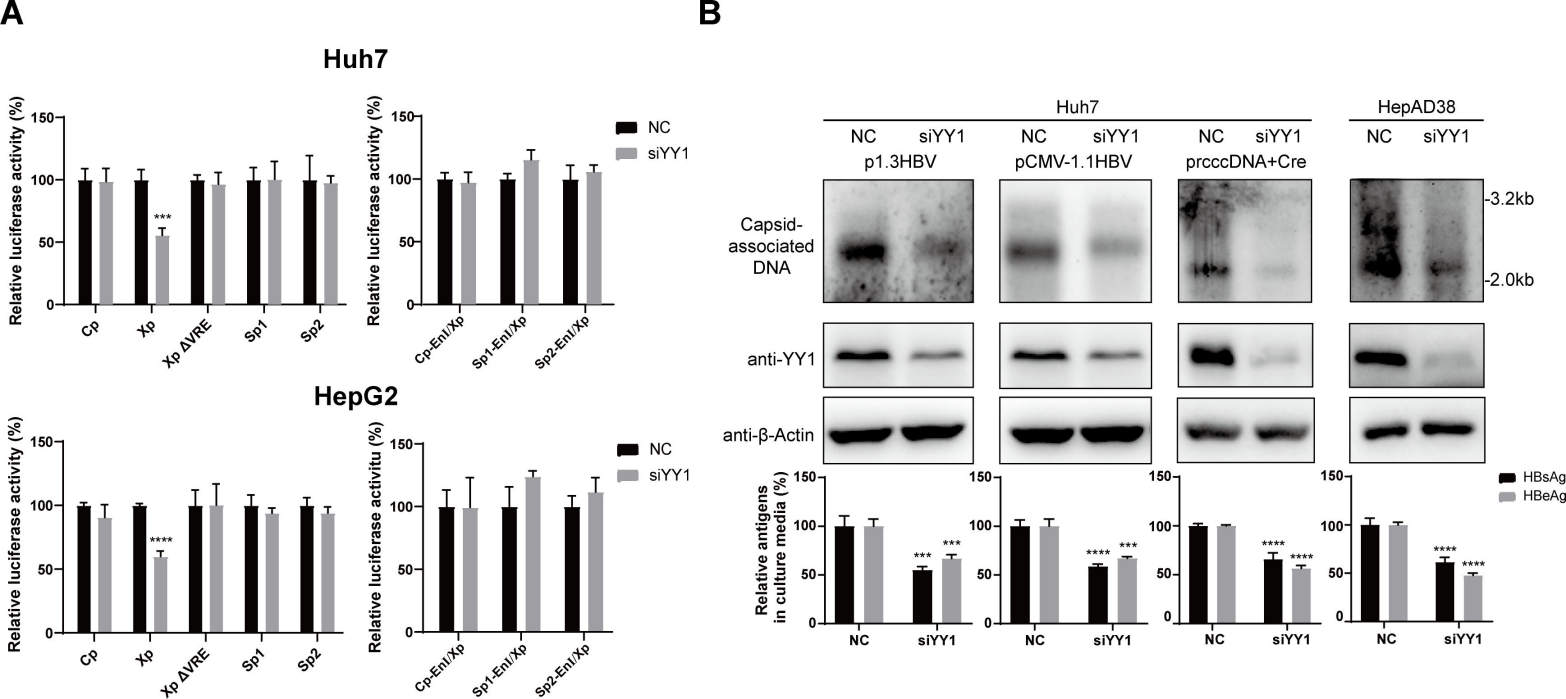
YY1 activates EnI/Xp promoter activity via VRE and upregulates HBV antigen expression and replication. (**A**) Effect of knockdown of endogenous YY1 on HBV promoter and EnI/Xp enhancer activities. Cells in 24-well plate were co-transfected with 0.6 µg indicated luciferase reporter plasmids and 0.1 µg Renilla expression plasmid. Media were changed 12 hours later, and 40 pmol chemically synthesized YY1-targeting siRNA (siYY1) or sequence scrambled control (siNC) was transfected. Relative luciferase activities were measured 2 days later using dual-luciferase reporter assay and calculated by normalizing against vector-transfected control group. Experiments were repeated at least three times. Group means and SDs were presented, and significances were calculated using two-way ANOVA test. ***, *P* < 0.001; ****, *P* < 0.0001. (**B**) Effect of knockdown of endogenous YY1 in HBV replication models. Huh7 cells in six-well plate were transfected with 2 µg p1.3HBV, 2 µg pCMV-1.1HBV, or 1 µg prcccDNA plus 1 µg pCre (left). Media were changed after 12 hours, and 80 pmol siNC or siYY1 was transfected. HepAD38 cells in 6 cm dishes were transfected with 240 pmol siNC or siYY1 (right). After 4 days, capsid-associated HBV DNA was analyzed in Southern blot, and HBV antigens in culture media were measured by ELISA.

We went on to test whether YY1 regulates HBV promoter/enhancer activities. Reporter assay showed that knockdown of endogenous YY1 expression only repressed EnI/Xp promoter activity but had no effect on its enhancer activity or activities of other promoter/enhancer elements (Fig. 8A). More importantly, knockdown of endogenous YY1 expression had no effect on the promoter activity of mutant EnI/Xp lacking VRE (Fig. 8A). It thus appeared that YY1 upregulates EnI/Xp promoter activity through VRE, and VCPIP1 participates in this process by enhancing binding of YY1 to VRE in EnI/Xp. Consistent with this observation, knockdown of endogenous YY1 expression resulted in reduced antigen expression and genome replication in plasmid- and rcccDNA-based HBV replication models (Fig. 8B).

**Fig 9:**
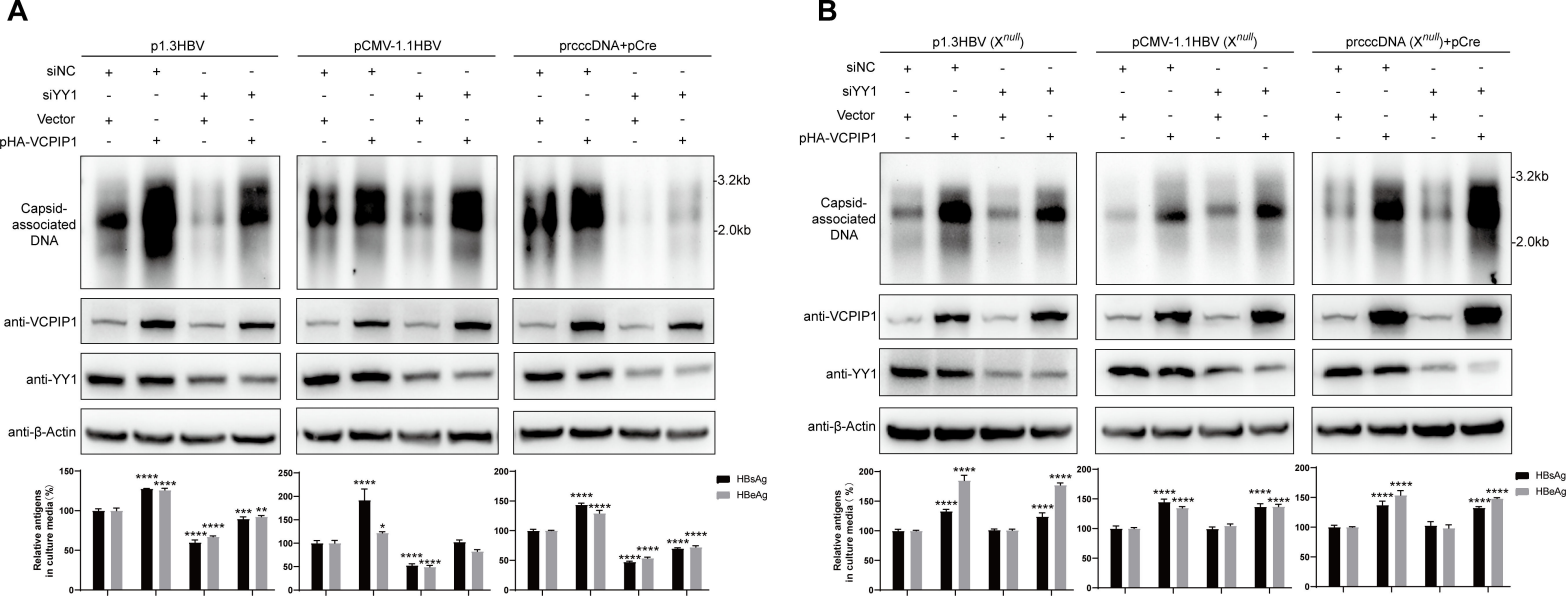
Effects of simultaneous YY1 knockdown and VCPIP1 overexpression on HBV antigen expression and replication. (**A**) Huh7 cells in six-well plate were transfected with 2 µg p1.3HBV, 2 µg pCMV-1.1HBV, or 1 µg prcccDNA plus 1 µg pCre (**A**), or 2 µg p1.3HBV, 2 µg pCMV-1.1HBV, or 1 µg prcccDNA plus 1 µg pCre (**B**) along with 1 µg pHA-VCPIP1 or vector control as indicated. Cells were changed into fresh media 12 hours later and transfected with 80 pmol chemically synthesized siYY1 or siNC as indicated. (**B**) Cells were similarly transfected as in (**A**), but corresponding X*^null^* mutant genome plasmids were used instead. Cells were harvested 4 days later, and capsid-associated HBV DNA was analyzed in Southern blot (top). Intracellular VCPIP1 and YY1 were analyzed in Western blot. Secreted HBsAg and HBeAg were measured using ELISA (bottom). Relative antigen levels are calculated by normalizing against vector/siNC-transfected control group. Experiments were repeated three times. Group means and SDs were presented, and significances were calculated using two-way ANOVA. *, *P* < 0.05; **, *P* < 0.01; ***, *P* < 0.001; ****, *P* < 0.0001.

Since YY1 upregulates only the promoter, but not enhancer, activity of EnI/Xp (Fig. 8A), YY1’s effects on HBV would have to involve transcription and expression of HBx. Indeed, knockdown of endogenous YY1 had minimal effects on mutant X*^null^* HBV genomes, in distinct contrast to wild-type genomes (Fig. 9). Furthermore, for both wild-type and HBx-deficient HBV genomes, VCPIP1 overexpression still displayed considerable upregulatory effects in the context of YY1 knockdown (Fig. 9). For wild-type HBV genome, this observation is most likely attributable to VCPIP1-mediated stabilization of HBx protein (Fig. 1F; Fig. S2). For the mutant X*^null^* HBV genomes, however, this result is consistent with reporter assay data showing VCPIP1’s effects on both promoter and enhancer activities of EnI/Xp (Fig. 5D; Fig. S4A) and confirmed that VCPIP1-mediated upregulation of EnI/Xp involves other mechanism(s) in addition to enhanced YY1 binding (Fig.S7 and S8).

## DISCUSSION

Two-hybrid screening and immunoprecipitation coupled with mass spectrometry have been extensively used for screening protein-protein interactions. Compared to these techniques, proximity labeling coupled with MS has the characteristic of identifying both stable and transient, direct and indirect binding partner(s) of the bait, as well as proteins that are only proximally localized with the bait (19). This is evident in our proximity labeling results obtained using a fusion protein between TurboID biotin ligase and HBx: directly HBx-binding DDB1, non-HBx-binding component of CRL4^DDB1^ E3 ligase complex (CUL4B), and components of CRL4^DDB1^-regulating CSN complex (COPS1-6,7A) made up one-third of identified proteins (Fig. 1B; Table S4). Among the rest of the identified proteins, we focused on VCPIP1 due to its known deubiquitinating enzyme (DUB) activity and demonstrated association both between HBx and endogenous/exogenous VCPIP1 and between *in vitro* translated HBx and VCPIP1, indicating a direct binding between the two (Fig. 1C and D). Some of the remaining proteins were also associated with HBx in preliminary co-IP testing (Fig. S9). Further work on these candidates could reveal additional host factors that might participate in HBx-related cellular and/or virological processes.

In the recent report by Wu et al., targeted yeast two-hybrid screening was performed on 74 known human DUBs using HBx as bait, and four DUBs were identified with potential HBx-binding capability, including VCPIP1 and COPS6 (17) identified in this work. Whether the interaction between CSN component COPS6 and HBx has any effect on CSN-mediated regulation of CRL4^DDB1^ activity or plays any role in HBx-mediated hijacking of CRL4^DDB1^ is an interesting possibility that is worth following up. Regarding VCPIP1, Wu et al. used co-IP and two-hybrid assay in yeast cells and mapped HBx-binding to VCPIP1’s C-terminal region downstream of Ubl domain (17). In agreement with this, our results showed that VCPIP1 mutants lacking the OTU domain (responsible for DUB activity) and the Ubl domain largely retained HBx-binding capacity (Fig. 1E).

VCPIP1’s stabilizing effect on HBx protein was studied both in the recent report by Wu et al. and in this work, and the results are mutually corroborating. Although VCPIP1-catalyzed removal of poly-ubiquitin chains from HBx leading to its rescue from ubiquitin-proteasomal degradation pathway seemed to be a straightforward and tempting explanation, Wu et al. showed that VCPIP1 does not affect HBx ubiquitination levels, and HBx mutant with all lysine residues changed to arginine is still stabilized by VCPIP1 (17), while we demonstrated that VCPIP1 mutant lacking the OTU domain still stabilizes HBx (Fig. 1F). Clearly, VCPIP1-mediated stabilizing of HBx protein does not depend on its DUB activity despite the fact that such stabilizing indeed rescues HBx from proteasomal degradation [Fig. S2D and reference (17)]. Mechanistic studies by Wu et al. demonstrated that VCPIP1 forms a tertiary complex with HBx and 26S proteasome regulatory subunit 6A (PSMC3) to prevent HBx degradation by 20S proteasome (17).

By using a series of transient or stable HBV replication models and HBV infection models based on HepG2-NTCP and PHH, we systematically and extensively characterized VCPIP1’s enhancing effects on HBV transcription, antigen production, and HBx expression in particular, as well as genome replication and progeny virus production (Fig. 2 and 3; Fig.S3). The recent report by Wu et al. also showed that overexpression of VCPIP1 and/or PSMC3 increased HBV transcription, antigen secretion, and progeny virus production in infected HepG2-NTCP cells (17). Although VCPIP1-mediated stabilizing of HBx protein is consistent with these observed effects, VCPIP1 mutant lacking OTU domain, which retained HBx-stabilizing capability (Fig. 1F), did not display the same level of upregulation in some replication models compared to wild-type VCPIP1 (Fig. 2A; Fig.S3B ). This suggested the involvement of mechanisms other than stabilization of HBx protein, and data obtained using mutant HBV genome replicons lacking HBx expression indeed proved that VCPIP1 was capable of HBx-independently upregulating HBV (Fig. 4).

By using reporter assay and DNA probe pull-down assay, we were able to show that VCPIP1 functions as positive regulator of one of HBV’s four *cis*-regulatory elements, EnI/Xp, and enhances both its promoter and enhancer activities through binding to a VRE mapped to −154 to −75 nt (Fig. 5 and 6). Details of VCPIP1’s association with VRE in EnI/Xp are still unclear at this stage, but the lack of direct binding between VRE probe and *in vitro* translated VCPIP1 (Fig. 6F) indicated the involvement of other directly DNA-binding factor(s). Interestingly, deletion of OTU domain did not affect VCPIP1’s association with VRE (Fig. 6E) but had different effects on promoter and enhancer activities of EnI/Xp (Fig. 5E). It would appear that the association of VCPIP1, with or without DUB activity, with VRE is sufficient for upregulating EnI/Xp-mediated transcription, but upregulation of EnI/Xp-mediated enhancement of distal promoter requires the OTU domain of VCPIP1 and/or its DUB activity.

The zinc finger protein YY1 is a ubiquitous transcription factor that, depending on context, activates or represses cellular and viral target genes through diverse mechanisms (32). Our results indicated that VCPIP1 enhances YY1 binding to VRE, both as dsDNA probe and in the context of HBV genome (Fig. 7; Fig. S8), which in turn upregulated EnI/Xp promoter activity but not its enhancer activity (Fig. 8). Details of the former process are not yet clear, as there was apparently no association between VCPIP1 and YY1 (Fig. S7). Moreover, the deletion of OTU domain did not affect VCPIP1’s association with VRE (Fig. 6E) but resulted in reduced YY1 binding to VRE (Fig. 7C). It is very likely that other cellular factors are involved in this process, and additional work is required to elucidate the underlying mechanisms.

The question whether the two non-overlapping putative YY1-binding sites within VRE (Fig. 7A) equally participate in YY1-mediated regulation of EnI/Xp promoter activity also remains to be addressed. Previously, a couple of putative YY1-binding sites in other parts of HBV genome have been studied. One such site located within Cp and close to direct repeat 1 (DR1) was associated with intramolecular recombination and chromosomal integration of HBV linear genome, but YY1 binding to this site was not demonstrated (33). By contrast, another study reported the binding of YY1 to the first YY1-binding site in VRE as characterized here and showed that such binding localized HBV rcccDNA close to a transcriptionally active chromosomal region in the nucleus and was associated with enhanced HBV transcription and antigen expression (34). More interestingly, YY1 was shown to associate with HBx in co-IP and HBx promoted YY1 recruitment to rcccDNA (34). Since DNA pull-down and reporter assays assessing YY1’s effects on EnI/Xp via VRE were performed in the absence of HBV genome or HBx (Fig. 7 and 8A), these earlier results were not sufficient to explain, but also not incompatible with, our observations. It is possible that YY1’s recruitment to HBV EnI/Xp via VRE has multiple effects through diverse mechanisms, while VCPIP1 could promote such recruitment both “directly” through YY1 and also indirectly through stabilizing HBx.

Since current evidences link binding of YY1 to VRE within EnI/Xp only to the latter’s promoter activity (Fig. 8), how VCPIP1 upregulates its enhancer activity (Fig. 5) is still unknown. The relevance of this aspect of VCPIP1-mediated upregulation was evident when VCPIP1 overexpression was shown to enhance antigen expression and genome replication from both wild-type and HBx-deficient HBV genomes even when endogenous YY1 expression was knocked down (Fig. 9). VRE-binding transcription factors not studied in details here due to apparent lack of response to VCPIP1 (Fig. 7) deserve further attention while addressing this issue. It is possible that VCPIP1 might modulate their activities without affecting their binding to VRE.

Transcriptional regulation through association with regulatory elements on DNA has not been previously reported for VCPIP1 and thus represents a rather novel function of VCPIP1. Whether such regulation also affects other cellular and viral genes, especially YY1 target genes, needs to be probed with further work. Meanwhile, the results presented here expand on the recently described HBx-stabilizing function of VCPIP1 and establish it as a multi-functional positive regulator of HBV.

## Data Availability

Data are available from the authors on request.

## SUPPLEMENTAL MATERIAL

Supplemental material jvi.01581-24-s0001.docx Tables S1 to S4; Figures S1 to S9.

ASM does not own the copyrights to Supplemental Material that may be linked to, or accessed through, an article. The authors have granted ASM a non-exclusive, world-wide license to publish the Supplemental Material files. Please contact the corresponding author directly for reuse.
