# Supplementary material for: Identification and characterization of host factor VCPIP1 as a multi-functional positive regulator of hepatitis B virus
Source: J Virol. 2024 Nov 4;98(12):e01581-24. doi: 10.1128/jvi.01581-24 (PMC11650987; doi:10.1128/jvi.01581-24)
Supplement: Supplemental material — Tables S1 to S4; Figures S1 to S9. [file jvi.01581-24-s0001.docx]

**Supplementary information**

**Table.S1 Primers, shRNAs and siRNAs used in this study**

| **Primers** | **Sequences（5’-3’）** |
| --- | --- |
| TB-F | TGACTCGAGCATGCATCTAGAGGG |
| TB-R | CTTTTCGGCAGACCGCAGACTGAT |
| deOTU-F | ATAAAAGGGGCTGCTTTGCCCAAAC |
| deOTU-R | CAGTAAGGCGCAATCGAACAGTTC |
| deUbl-F | ATTCTAAAAAGTAAAGCTGAAGGTG |
| deUbl-R | CTCCTTAGAAGTTGTCGGTGAATAG |
| Xnull-F | GCTAACTGGATCCTGCGCGGGACGT |
| Xnull-R | AGCACAGCCTAGCAGCCATGGAAAC |
| Xp-F | GATCCGGAAACTTCCTGTAAACAGGCCTATTGATTGGAAAGTATGTCA |
| Xp-R | TTGAAGGCTCTCAAGGGCGAATTCATGGAAAGGATGTATACTTGCGGG |
| -404-R | CCTGTTTACAGGAAGTTTCCTAAA |
| -354-R | GCAAACCCCAAAAGACCCACAATA |
| -325-F | TTTAATGCCTTTATATGCATGTATAC |
| -304-R | ATGCATATAAAGGCATTAAAGCAGG |
| -275-F | CGCCAACTTACAAGGCCTTTCTAA |
| -254-R | AAAGGCCTTGTAAGTTGGCGAGAAA |
| -225-F | GTTGCTCGGCAACGGCCTGGTCT |
| -204-R | CCAGGCCGTTGCCGAGCAACG |
| -175-F | CACTGGTTGGGGCTTGGCCATAGG |
| -154-R | TGGCCAAGCCCCAACCAGTGGGGGTT |
| -125-F | TGTCTCCTCTGCCGATCCATACTGC |
| -100-R | ATGGATCGGCAGAGGAGACACAAAG |
| -75-F | CACAGCAGGTCTGGGGCAAAACTCA |
| -50-R | TTTGCCCCAGACCTGCTGTGAGCAA |
| +1-F | CTCCCGCAAGTATACATCCTTTCCA |
| 18S-F | CAGCCACCCGAGATTGAGCA |
| 18S-R | TAGTAGCGACGGGCGGTGTG |
| pgAD-F | GCCTTAGAGTCTCCTGAGCA |
| pgBC-F | GCCTTAGAGTCTCCGGAACA |
| pg-R | GAGGGAGTTCTTCTTCTAGG |
| TR-F | ACCGACCTTGAGGCATACTT |
| TR-R | GCCTACAGCCTCCTAGTACA |
| CEBP/β-F | CTGGAGACGCAGCACAAG |
| CEBP/β-R | ACAGCTGCTCCACCTTCTTC |
| GR-F | ACAGCATCCCTTTCTCAACAG |
| GR-R | AGATCCTTGGCACCTATTCCAAT |
| NRF1-F | GCTGATGAAGACTCGCCTTCT |
| NRF1-R | TACATGAGGCCGTTTCCGTTT |
| STAT4-F | GCTTAACAGCCTCGATTTCAAGA |
| STAT4-R | GAGCATGGTGTTCATTAACAGGT |
| YY1-F | ACGGCTTCGAGGATCAGATTC |
| YY1-R | TGACCAGCGTTTGTTCAATGT |
| shVCPIP1-F | CCGGGAAAGTTGTCCACACTATATTCTCGAGAATATAGTGTGGACAACTTTCTTTTTG |
| shVCPIP1-R | AATTCAAAAAGAAAGTTGTCCACACTATATTCTCGAGAATATAGTGTGGACAACTTTC |
| siYY1- sense | CAUAAAGGCUGCACAAAGATT |
| siYY1-antisense | UCUUUGUGCAGCCUUUAUGTT |
| siNC-sense | UUCUCCGAACGUGUCACGUTT |
| siNC-antisense | ACGUGACACGUUCGGAGAATT |
| rcccHBV-YY1-F | GTTTGCTGACGCAACCCCCA |
| rcccHBV-YY1-R | CCTGCTGCGAGCAAAACAAG |

**Table.S2 MaxQuant parameters used in this study**

| **Parameter** | **Value** |
| --- | --- |
| Version | 2.1.3.0 |
| Include contaminants | TRUE |
| PSM FDR | 0.01 |
| PSM FDR Crosslink | 0.01 |
| Protein FDR | 0.01 |
| Site FDR | 0.01 |
| Use Normalized Ratios For Occupancy | TRUE |
| Min. peptide Length | 7 |
| Min. score for unmodified peptides | 0 |
| Min. score for modified peptides | 40 |
| Min. delta score for unmodified peptides | 0 |
| Min. delta score for modified peptides | 6 |
| Min. unique peptides | 0 |
| Min. razor peptides | 1 |
| Min. peptides | 1 |
| Use only unmodified peptides and | TRUE |
| Modifications included in protein quantification | Oxidation (M); Acetyl (Protein N-term) |
| Peptides used for protein quantification | Razor |
| Discard unmodified counterpart peptides | TRUE |
| Label min. ratio count | 2 |
| Use delta score | FALSE |
| iBAQ | TRUE |
| iBAQ log fit | TRUE |
| Match between runs | TRUE |
| Matching time window [min] | 0.7 |
| Match ion mobility window [indices] | 0.05 |
| Alignment time window [min] | 20 |
| Alignment ion mobility window [indices] | 1 |
| Find dependent peptides | TRUE |
| Dependent peptide FDR | 0.01 |
| Mass bin size | 0.0065 |
| Decoy mode | revert |
| Include contaminants | TRUE |
| Advanced ratios | FALSE |
| Second peptides | TRUE |
| Stabilize large LFQ ratios | TRUE |
| Separate LFQ in parameter groups | TRUE |
| Require MS/MS for LFQ comparisons | TRUE |
| Calculate peak properties | FALSE |
| Main search max. combinations | 200 |
| Advanced site intensities | TRUE |
| Write msScans table | FALSE |
| Write msmsScans table | TRUE |
| Write ms3Scans table | TRUE |
| Write allPeptides table | TRUE |
| Write mzRange table | TRUE |
| Write DIA fragments table | FALSE |
| Write DIA fragments quant table | FALSE |
| Write pasefMsmsScans table | TRUE |
| Write accumulatedMsmsScans table | TRUE |
| Max. peptide mass [Da] | 4600 |
| Min. peptide length for unspecific search | 8 |
| Max. peptide length for unspecific search | 25 |
| Razor protein FDR | TRUE |
| Disable MD5 | FALSE |
| Max mods in site table | 3 |
| Match unidentified features | FALSE |
| Evaluate variant peptides separately | TRUE |
| Variation mode | None |
| MS/MS tol. (FTMS) | 20 ppm |
| Top MS/MS peaks per Da interval. (FTMS) | 12 |
| Da interval. (FTMS) | 100 |
| MS/MS deisotoping (FTMS) | TRUE |
| MS/MS deisotoping tolerance (FTMS) | 7 |
| MS/MS deisotoping tolerance unit (FTMS) | ppm |
| MS/MS higher charges (FTMS) | TRUE |
| MS/MS water loss (FTMS) | TRUE |
| MS/MS water loss (FTMS for cross link) | FALSE |
| MS/MS ammonia loss (FTMS) | TRUE |
| MS/MS ammonia loss (FTMS for cross link) | FALSE |
| MS/MS dependent losses (FTMS) | TRUE |
| MS/MS recalibration (FTMS) | FALSE |
| MS/MS tol. (ITMS) | 0.5 Da |
| Top MS/MS peaks per Da interval. (ITMS) | 8 |
| Da interval. (ITMS) | 100 |
| MS/MS deisotoping (ITMS) | FALSE |
| MS/MS deisotoping tolerance (ITMS) | 0.15 |
| MS/MS deisotoping tolerance unit (ITMS) | Da |
| MS/MS higher charges (ITMS) | TRUE |
| MS/MS water loss (ITMS) | TRUE |
| MS/MS water loss (ITMS for cross link) | FALSE |
| MS/MS ammonia loss (ITMS) | TRUE |
| MS/MS ammonia loss (ITMS for cross link) | FALSE |
| MS/MS dependent losses (ITMS) | TRUE |
| MS/MS recalibration (ITMS) | FALSE |
| MS/MS tol. (TOF) | 25 ppm |
| Top MS/MS peaks per Da interval. (TOF) | 16 |
| Da interval. (TOF) | 100 |
| MS/MS deisotoping (TOF) | TRUE |
| MS/MS deisotoping tolerance (TOF) | 0.01 |
| MS/MS deisotoping tolerance unit (TOF) | Da |
| MS/MS higher charges (TOF) | TRUE |
| MS/MS water loss (TOF) | TRUE |
| MS/MS water loss (TOF for cross link) | FALSE |
| MS/MS ammonia loss (TOF) | TRUE |
| MS/MS ammonia loss (TOF for cross link) | FALSE |
| MS/MS dependent losses (TOF) | TRUE |
| MS/MS recalibration (TOF) | FALSE |
| MS/MS tol. (Unknown) | 20 ppm |
| Top MS/MS peaks per Da interval. (Unknown) | 12 |
| Da interval. (Unknown) | 100 |
| MS/MS deisotoping (Unknown) | TRUE |
| MS/MS deisotoping tolerance (Unknown) | 7 |
| MS/MS deisotoping tolerance unit (Unknown) | ppm |
| MS/MS higher charges (Unknown) | TRUE |
| MS/MS water loss (Unknown) | TRUE |
| MS/MS water loss (Unknown for cross link) | FALSE |
| MS/MS ammonia loss (Unknown) | TRUE |
| MS/MS ammonia loss (Unknown for cross link) | FALSE |
| MS/MS dependent losses (Unknown) | TRUE |
| MS/MS recalibration (Unknown) | FALSE |
| Site tables | GlyGly(K)Sites.txt;Oxidation (M)Sites.txt |

**Table.S3 Antibodies used in this study**

| **Antibodies** | **Company** |
| --- | --- |
| Anti-HBx | Abmart |
| Anti-HA（M20003） | Abmart |
| Anti-β-Actin（A3854） | Sigma |
| Anti-mouse/rabbit IgG（H+L）HRP | Invitrogen |
| Streptavidin-HRP | Thermo |
| Anti-VCPIP1（#88153） | CST |
| Anti-VCPIP1（GTX107169） | GeneTex |
| Anti-FLAG（F3165） | Sigma |
| Anti-Digoxigenin Fab fragments | Roche |
| Anti-GR（#12041） | CST |
| Anti-NRF1（ab175932） | Abcam |
| Anti-STAT4（13028-1-AP） | Proteintech |
| Anti-CEBP/β（ab32358） | Abcam |
| Anti-YY1（ab109237） | Abcam |
| Anti-YY1（#46395） | CST |

**Table.S4 Identified proteins in** **LC-MS/MS analysis**

| **Uniprot** | **Proteins** | **LogFC** |
| --- | --- | --- |
| FLAG-HBx | FLAG-HBx | 12.746769 |
| Q13098 | GPS1 (COPS1) | 5.3876144 |
| Q92905 | COPS5 | 5.1281557 |
| Q9UNS2 | COPS3 | 4.7166373 |
| P22695 | UQCRC2 | 4.5181628 |
| Q96JH7 | VCPIP1 | 4.4593085 |
| Q13620 | CUL4B | 4.4333311 |
| Q7L5N1 | COPS6 | 4.3592378 |
| P61803 | DAD1 | 4.0875285 |
| Q9NZT1 | CALML5 | 4.0553058 |
| O14929 | HAT1 | 3.8981123 |
| Q9BT78 | COPS4 | 3.893394 |
| Q9UBW8 | COPS7A | 3.8640154 |
| O95372 | LYPLA2 | 3.7837991 |
| Q9UNE7 | STUB1 | 3.7285564 |
| Q9BWG6 | SCNM1 | 3.3043777 |
| P24539 | ATP5PB | 3.1632085 |
| O14757 | CHEK1 | 3.0546182 |
| Q02978 | SLC25A11 | 2.9391099 |
| Q92733 | PRCC | 2.8958655 |
| P61201 | COPS2 | 2.859743 |
| Q8WVJ2 | NUDCD2 | 2.7677987 |
| Q6W2J9 | BCOR | 2.7439544 |
| Q14814 | MEF2D | 2.6992512 |
| Q13123 | IK | 2.6265153 |
| Q9Y314 | NOSIP | 2.6100143 |
| Q6IQ49 | SDE2 | 2.3346471 |
| Q7L590 | MCM10 | 2.3193726 |
| Q96S55 | WRNIP1 | 2.281955 |
| P50990 | CCT8 | 2.2485342 |
| Q16531 | DDB1 | 2.109347 |


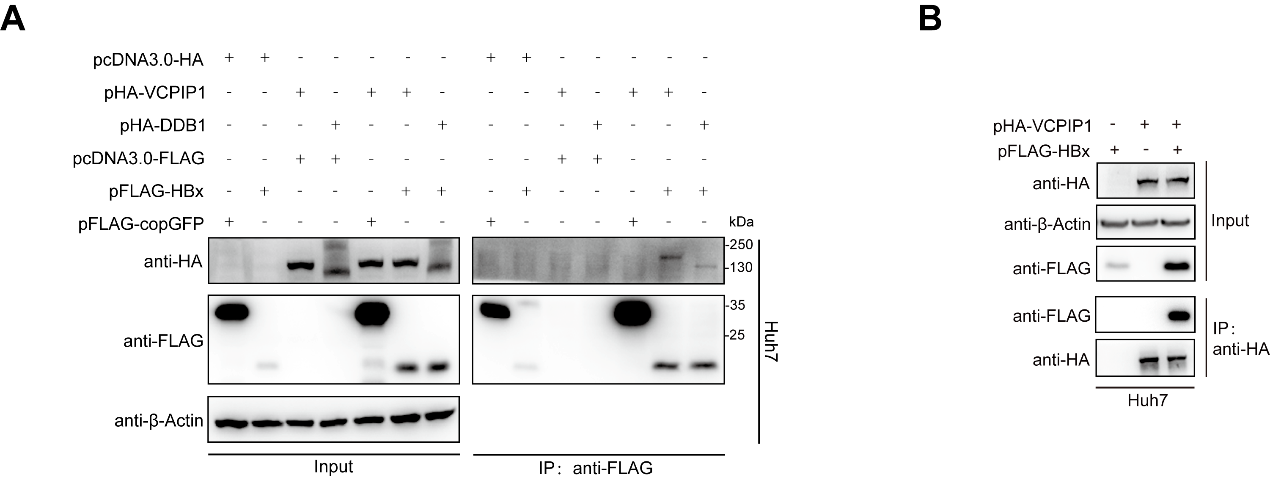


**Fig.S1 Co-immunoprecipitation of VCPIP1 and known HBx-binder DDB1 with HBx.**

**(A)** Cells in 6 cm dishes were transfected with 3 μg pFLAG-copGFP, pFLAG-HBx, pHA-VCPIP1 or pHA-DDB1 plus 3 μg vector control as indicated, or 3 μg pFLAG-copGFP plus 3 μg pHA-VCPIP1, 3 μg pFLAG-HBx plus 3 μg pHA-VCPIP1 or 3 μg pFLAG-HBx plus 3 μg pHA-DDB1. **(B)** Cells in 6 cm dishes were transfected with 3 μg pFLAG-HBx plus 3 μg pHA-VCPIP1 or vector control as indicated. Two days later, FLAG-tagged HBx and HA-tagged VCPIP1 in cell lysates was captured using anti-FLAG and anti-HA magnetic beads respectively, and co-immunoprecipitated proteins were analyzed in Western blot using indicated antibodies.

**
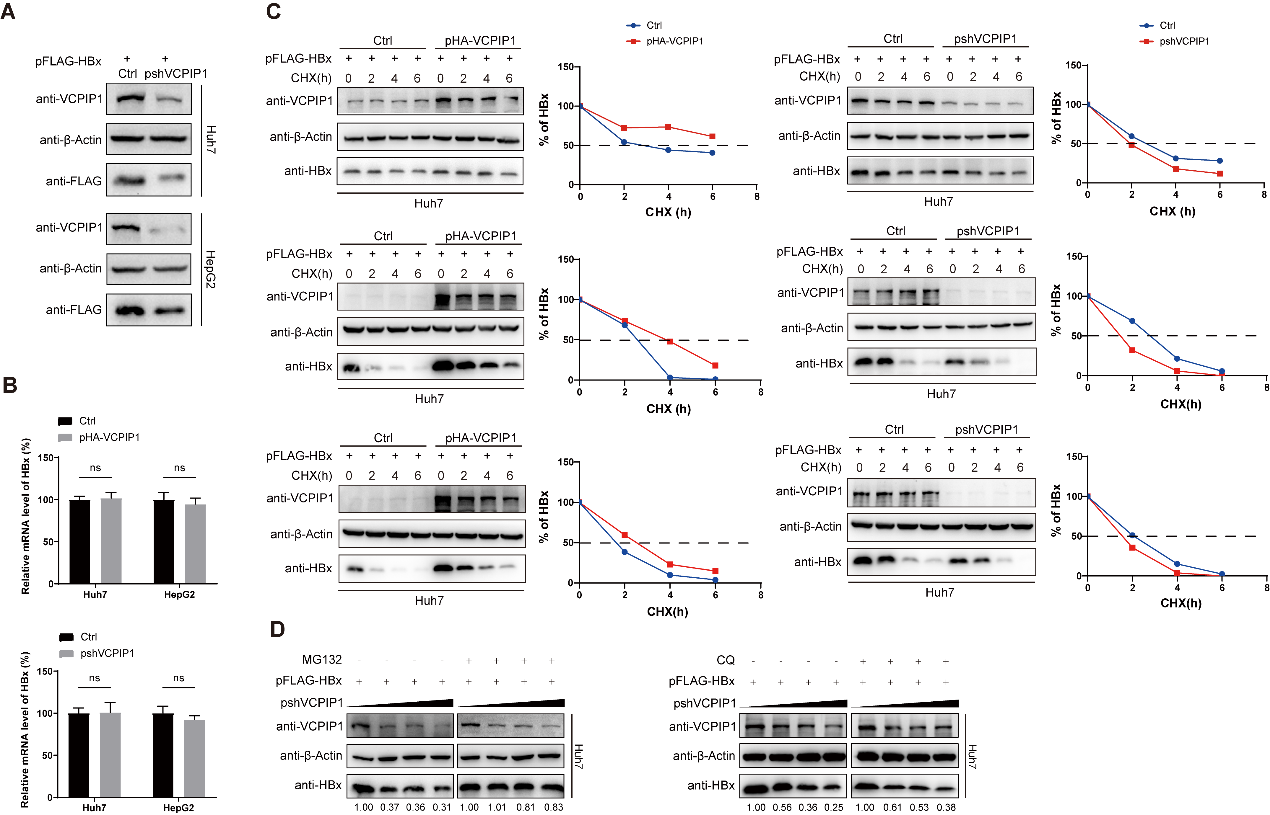
**

**Fig.S2 VCPIP1 improves HBx protein stability**

(**A**) Effect of knockdown of endogenous VCPIP1 on HBx protein level. Cells in 12-well plate were transfected with 1 μg pshVCPIP1 and 1 μg pFLAG-HBx. Cells were lysed 2 days later and VCPIP1 and HBx protein levels were analyzed in Western blot. (**B**) Effect of VCPIP1 on HBx mRNA level. Cells in 12-well plate were transfected with 1 μg pFLAG-HBx and 1 μg pHA-VCPIP1 or pshVCPIP1 as indicated. After 2 days, intracellular RNA was extracted and HBx mRNA levels were measured using RT-qPCR. Relative RNA levels are calculated by normalizing against vector-transfected control group. (**C**) Effect of VCPIP1 on HBx protein degradation. Huh7 cells in 12-well plate were transfected with 1 μg pFLAG-HBx and 1 μg pHA-VCPIP1 or pshVCPIP1 as indicated. After 2 days, cells were changed into fresh media supplemented with 10 μM CHX and sampled at 0, 2, 4, and 6 hours. HBx protein levels were analyzed in Western blot and signals were quantified by densitometry scanning, followed by normalization against vector-transfected control group (right). (**D**) Effect of VCPIP1 on HBx protein degradation in the presence of proteasome inhibitor MG132 and lysosome inhibitor chloroquine (CQ). Huh7 cells in 12-well plate were co-transfected with 1 μg pFLAG-HBx and 0, 0.25, 0.5 or 1 μg of pshVCPIP1. After 2 days, cells were treated with 10 μM MG132 or CQ for 6 hours before harvest. Cells were lysed and protein levels of VCPIP1 and HBx were analyzed in Western blot. HBx protein levels were quantified by densitometry scanning and normalized against vector-transfected control group. Group means and SDs were presented and significances were calculated using two-way ANOVA test; ns, *P*>0.05.

**
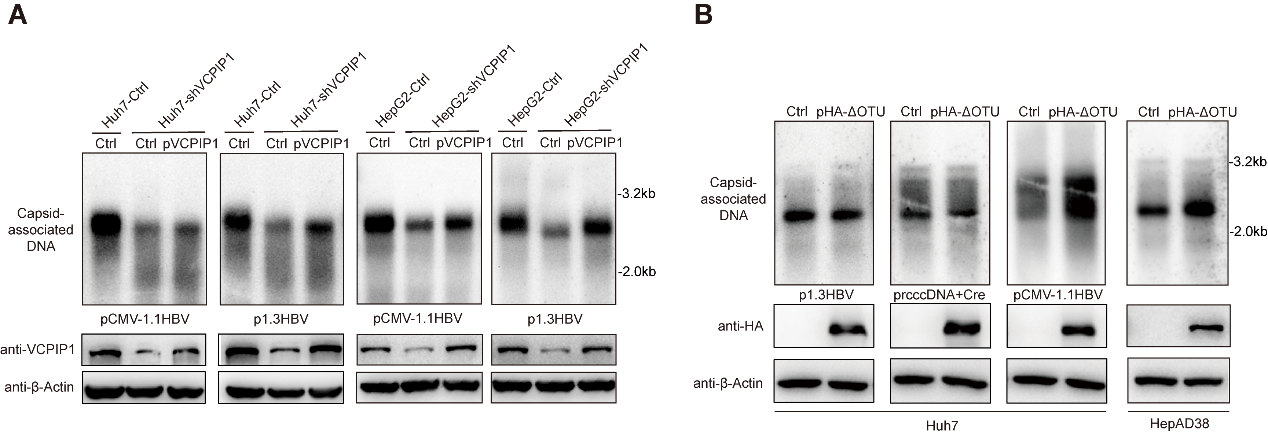
**

**Fig.S3 Effect of VCPIP1 in HBV replication models**

(**A**) Effect of rescued VCPIP1 expression on HBV replication in cells with stable knockdown of endogenous VCPIP1. Cells stably transducted with shVCPIP1 were transfected with 2 μg p1.3HBV or pCMV-1.1HBV, along with 1 μg pHA-VCPIP1 or vector control as indicated. After 4 days, intracellular capsid-associated HBV DNA was analyzed in Southern blot and VCPIP1 protein levels were analyzed in Western blot. (**B**) Effect of overexpressed VCPIP1 mutant with OTU domain deleted on HBV replication. Huh7 cells in 6-well plate were transfected with 2 μg p1.3HBV, 2 μg pCMV-1.1HBV, or 1 μg prcccDNA plus 1 μg pCre, along with 1 μg pHA-VCPIP1ΔOTU. HepAD38 cells in 6 cm dishes were transfected with 6 μg pHA-VCPIP1ΔOTU. After 4 days, capsid-associated HBV DNA was analyzed in Southern blot and VCPIP1ΔOTU protein levels were analyzed in Western blot.


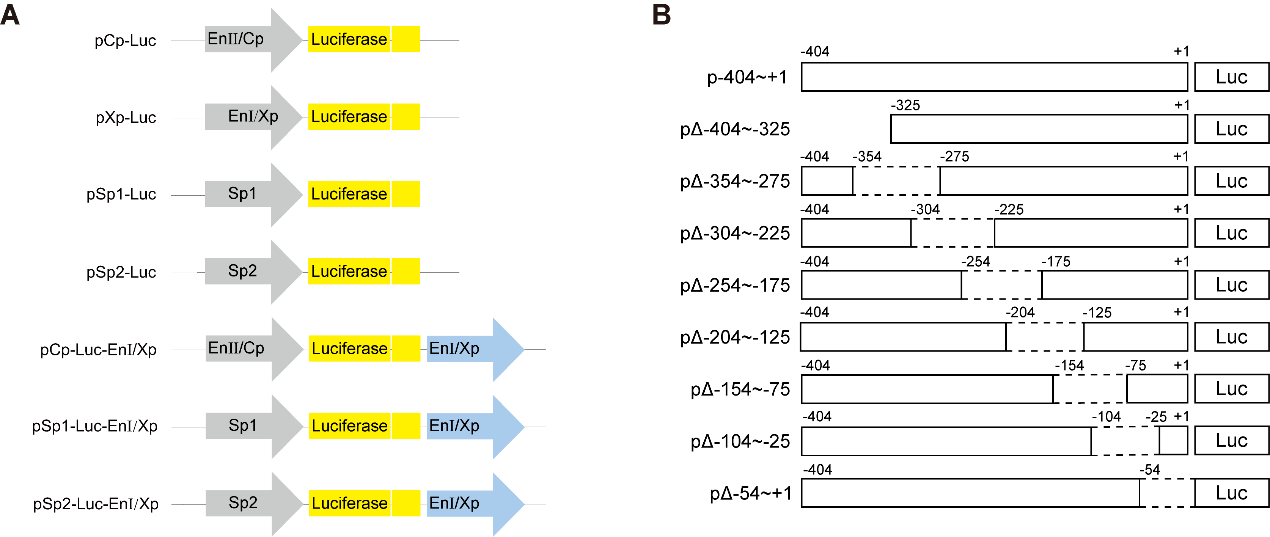


**Fig.S4 Schematic representation of HBV enhancer/promoter reporter constructs**

(**A**) Constructs of HBV enhancer/promoter reporters and reporters using EnI/Xp as enhancer. (**B**) Serially overlapping deletions within EnI/Xp used for mapping VCPIP1 responsive element (VRE).


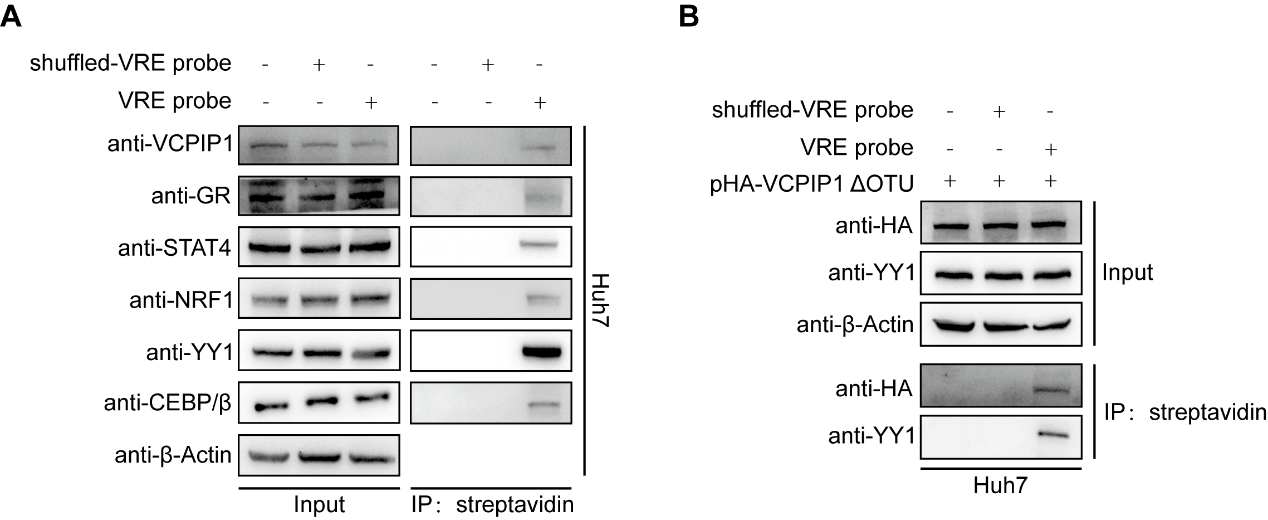


**Fig.S5 Binding of VRE by VCPIP1 and preidicted VRE-binding transcription factors**

Chemically synthesized biotin-labeled VRE double stranded DNA probe or control probe with the sequences shuffled to destroy predicted binding sites were used to capture endogenous VCPIP1 and predicted VRE-binding transcription factors **(A)**, or exogenous VCPIP1 ΔOTU and YY1 **(B)** in cell lysates in DNA pull-down assay using streptavidin beads. Captured protein was detected using Western blot. Shuffled-VRE probes were prepared by annealling biotin-5’-TTTTACTGCTGTCGATGTCCGTTCGGCCTCCGACCACAGTATGT

CCCCGACGTGTGTGTGACTCGTTACTAGGCCCTACA-3’ and unlabrlled reverse complement oligo.


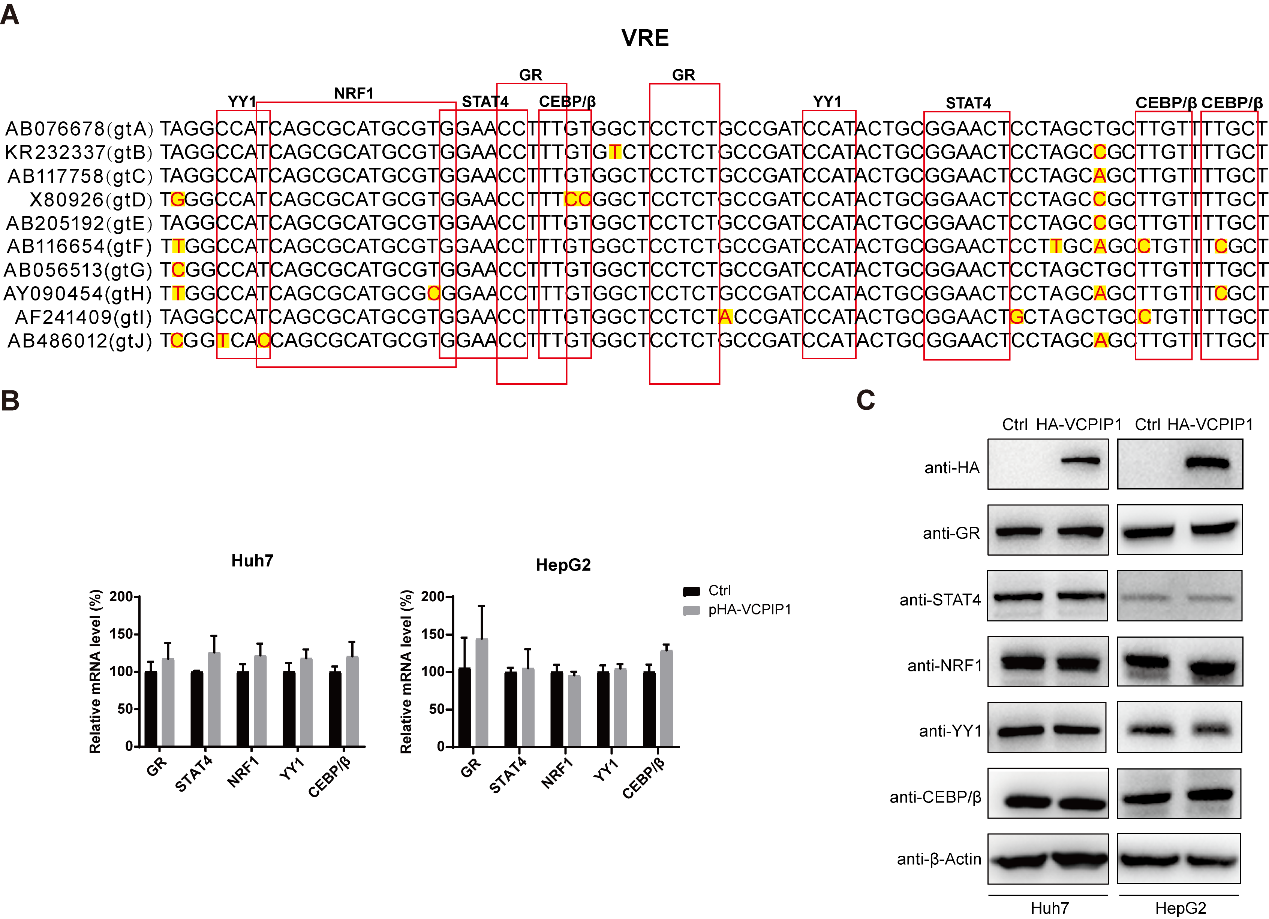


**Fig.S6 VCPIP1 does not affect mRNA or protein levels of predicted VRE-binding transcription factors**

(**A**) Alignment of VRE sequences of representative strains of currently recognized HBV genotypes (A–J) with predicted transcription factor binding sites indicated. Effects of VCPIP1 overexpression on transcription factor transcription (**B**) and protein expression (**C**). Cells in 12-well plate were transfected with 2 μg vector or pHA-VCPIP1. After 2 days, intracellular mRNA levels of predicted VRE-binding transcription factors were measured in RT-qPCR and normalized against vector control. Protein levels were analyzed in Western blot (**C**).


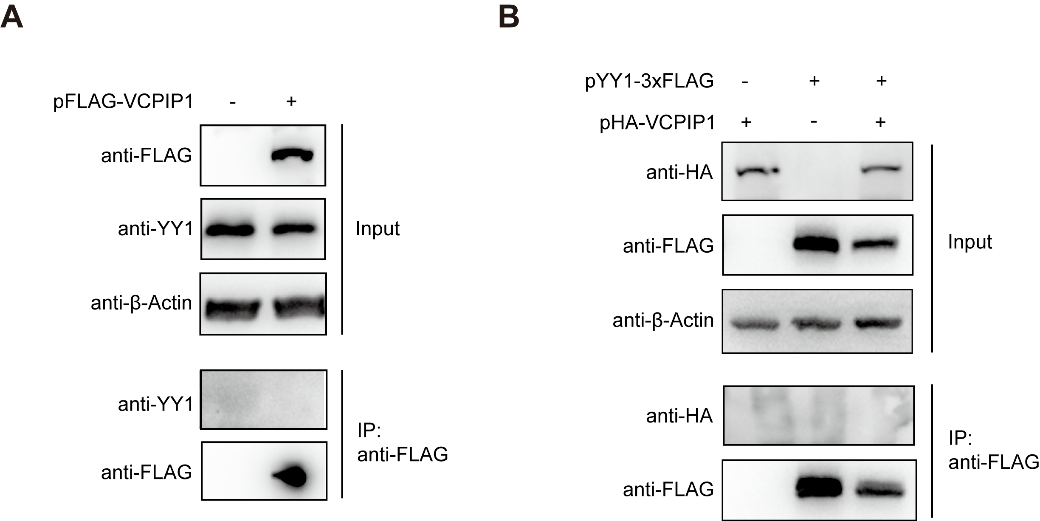


**Fig.S7 VCPIP1 shows no association with endogenous or exogenous YY1 in co-IP assay**

(**A**) Huh7 cells in 6 cm dish were transfected with 6 μg pFLAG-VCPIP1. After 2 days, cells were lysed for co-IP assay using FLAG antibody for immunoprecipitation. (**B**) Huh7 cells in 6 cm dish were transfected with pHA-VCPIP1 and/or 3 μg pYY1-3×FLAG as indicated. After 2 days, cells were lysed for co-IP assay using FLAG antibody for immunoprecipitation.


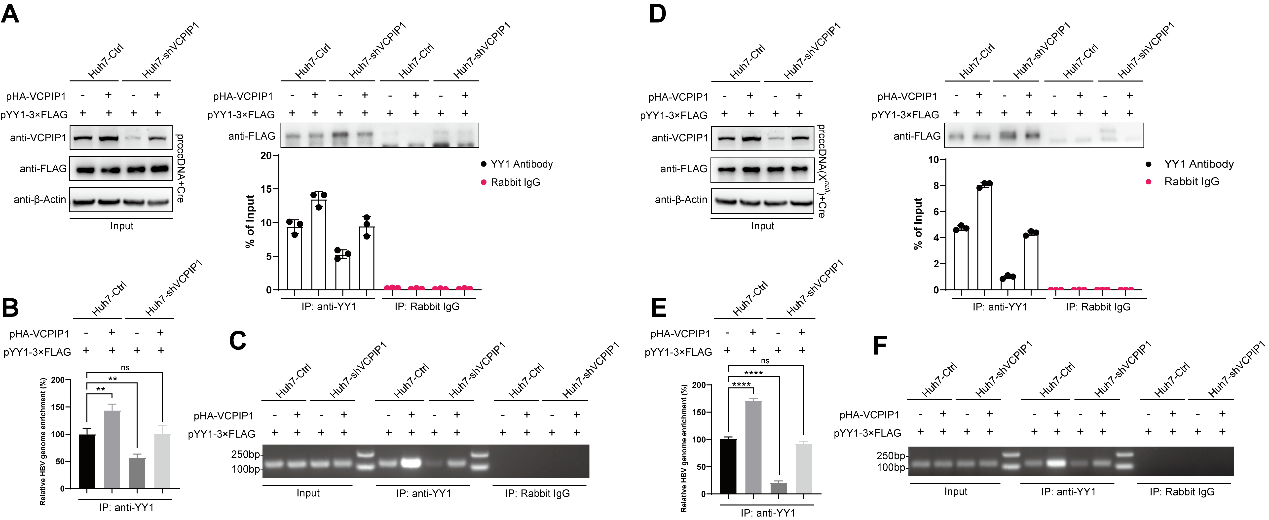


**Fig.S8 Effect of VCPIP1 on YY1 occupancy at VRE on HBV rcccDNA**

Huh7 cells stably transducted with shVCPIP1 or shCtrl were transfected with 3 μg prcccDNA (**A-C**) or prcccDNA(X*^null^*) (**D-F**) plus 3 μg pCre, along with 3 μg pYY1-3×FLAG and 1 μg pHA-VCPIP1 or vector control as indicated. Four days after transfection, cells were subjected to ChIP assay. VCPIP1 and YY1 protein were detected by Western Blot, and rcccDNA immunoprecipitated by YY1 antibody or control Rabbit IgG was analyzed in PCR (**C/F**), and quantitated in qPCR and presented as percentage of input (**A/D**). Relative rcccDNA enrichment were measured using qPCR, and calculated by normalizing against vector transfected shCtrl group (**B/E**). Experiments were repeated at least three times. Group means and SDs were presented and significances were calculated using one-way ANOVA. **, *P*<0.01; ****, *P*<0.0001; ns, *P*>0.05.


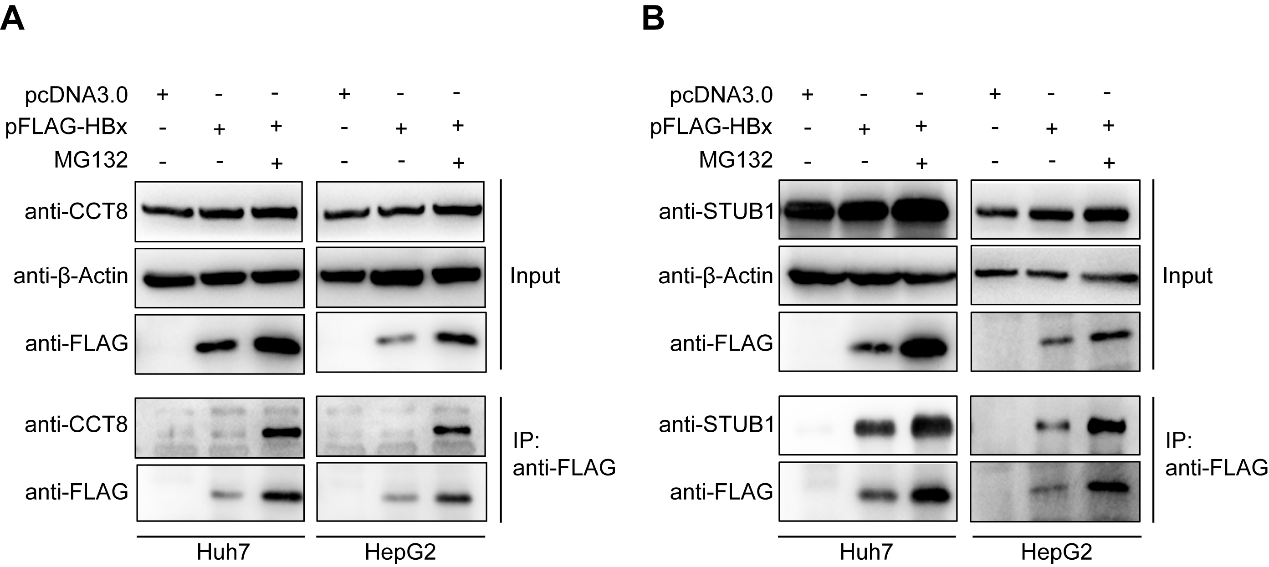


**Fig.S9 Co-immunoprecipitation of endogenous CCT8 and STUB1 with HBx.**

Cells in 6 cm dishes were transfected with 6 μg pFLAG-HBx or 6 μg vector control as indicated. After 2 days, cells were treated with 10 μM MG132 or DMSO as indicated for 6 hours before harvest. FLAG-tagged HBx in cell lysates was captured using anti-FLAG magnetic beads and co-immunoprecipitated CCT8 **(A)** /STUB1 **(B)** were analyzed in Western blot using indicated antibodies.
